# Prevalence of Drug-Resistant Tuberculosis in Sudan: A Systematic Review and Meta-Analysis

**DOI:** 10.3390/antibiotics10080932

**Published:** 2021-07-31

**Authors:** Khalid Hajissa, Mahfuza Marzan, Mubarak Ibrahim Idriss, Md Asiful Islam

**Affiliations:** 1 Department of Medical Microbiology & Parasitology, School of Medical Sciences, Universiti Sains Malaysia, Kubang Kerian 16150, Kelantan, Malaysia; khalidhaj@usm.my; 2 Department of Zoology, Faculty of Science and Technology, Omdurman Islamic University, P.O. Box 382, Omdurman 14415, Sudan; 3 Department of Microbiology, Jahangirnagar University, Savar, Dhaka 1342, Bangladesh; mmarzan@juniv.edu; 4 Laboratory Division, Kassala State Ministry of Health, Kassala, Sudan; mubarak.mareh@yahoo.com; 5 Department of Haematology, School of Medical Sciences, Universiti Sains Malaysia, Kubang Kerian 16150, Kelantan, Malaysia

**Keywords:** tuberculosis, antibiotic resistance, drug-resistant, prevalence, epidemiology, Sudan, systematic review, meta-analysis

## Abstract

Drug-resistant tuberculosis (DR-TB) is still one of the most critical issues impeding worldwide TB control efforts. The aim of this systematic review and meta-analysis was to give an updated picture of the prevalence of DR-TB in Sudan. A comprehensive systematic search was performed on four electronic databases (PubMed, Scopus, Web of Science and Google Scholar) to identify all published studies reporting prevalence data of DR-TB in Sudan. Sixteen eligible studies published during 2002–2020 were included. Using meta-analysis of proportions, the pooled prevalence of TB cases with resistance to any anti-TB drugs was 47.0% (95% CI: 35.5–58.6%). The overall prevalence of mono, multi, poly and extensive drug resistance were estimated to be 16.2% (95% CI: 9.0–23.4%), 22.8% (95% CI: 16.0–29.7%), 6.8% (95% CI: 0.5–13.0%) and 0.7% (95% CI: 0–2.1%), respectively. Considering any first-line anti-TB drugs, the resistance prevalence was highest for isoniazid (32.3%) and streptomycin (31.7%), followed by rifampicin (29.2%). In contrast, resistance against second-line drugs was reported for only two antibiotics, namely, ofloxacin (2.1%) and kanamycin (0.7%). Of note, the resistance profile of the previously treated patients was found to be remarkably high compared with the newly diagnosed TB patients. The relatively high prevalence estimation of anti-TB drug resistance warrants strengthening TB control and treatment strategies in Sudan.

## 1. Introduction

Despite the improvements in case identification, cure rates and implementation of a widely adopted control strategy, tuberculosis (TB) remains a significant health threat and continues to be one of the top infectious and fatal diseases [1,2]. The global TB statistics revealed 10 million infected individuals and approximately 1.4 million deaths in 2019 worldwide [3]. The disease geographic distribution disproportionately varies within countries and across the globe, and poverty is the strongest predictor of incidence [4,5]. In Africa, where inadequate diagnosis and treatment are extremely common, the incidence rate of the disease is particularly high and accounts for 25% of the global TB cases [6]. Sudan is among the developing countries where TB is a major public health challenge, with an estimated 29,000 cases in 2019 [3].

The prevention of new TB infections and effective treatment of established ones are critical to achieving remarkable reductions in the burden of the disease and associated deaths [7]. Globally, the successful treatment rate among TB-confirmed, -reported and -treated cases in 2018 was 85% [1]. However, the inadequate treatment of TB-infected patients enables bacterial strains to develop several resistance strategies against antimicrobial agents despite effectively killing the majority of the invaded bacteria [8]. Current efforts for accelerated TB control are challenged by the emergence of antibiotic resistance resulting in drug-resistant tuberculosis (DR-TB) [9]. The World Health Organisation (WHO) estimated that 465,000 of TB cases in 2019 had rifampicin resistance, of which 78% were further confirmed as multidrug-resistant TB (MDR-TB), having TB strains resistant to rifampicin and isoniazid [3]. Proper combinations of first- and second-line TB drugs have been effectively used in treating patients with MDR-TB, but the emergence of extensively drug-resistant tuberculosis (XDR-TB), which was defined as MDR-TB with resistance to fluoroquinolone, and at least one of the injectable second-line drug, has caused recent concern [9,10,11]. The increasing incidence of MDR-TB and XDR-TB in many parts of the world further threatens TB control efforts due to the high risk of treatment failure and death.

TB drug-resistance patterns in a country always reflect the effectiveness of its current and prior TB control programs. Therefore, regular surveillance of TB drug resistance is central to combating the global burden of TB and preventing the wide spread of antimicrobial resistance [12]. However, performing surveillance in most of the African countries is often limited by a lack of resources. This situation is reflected by the absence or insufficient data on anti-TB drug resistance [13]. Therefore, urgent efforts are needed to determine the true burden of DR-TB throughout Africa [14]. In Sudan, the rate of DR-TB has been reported in several published studies; however, most of these reports presented local information or region-specific data, and no study has systematically evaluated DR-TB prevalence data in Sudan. Therefore, this systematic review and meta-analysis (SRMA) was conducted to provide an updated and comprehensive assessment of the burden of DR-TB in Sudan.

## 2. Results

### 2.1. Study Selection

**Figure 1:**
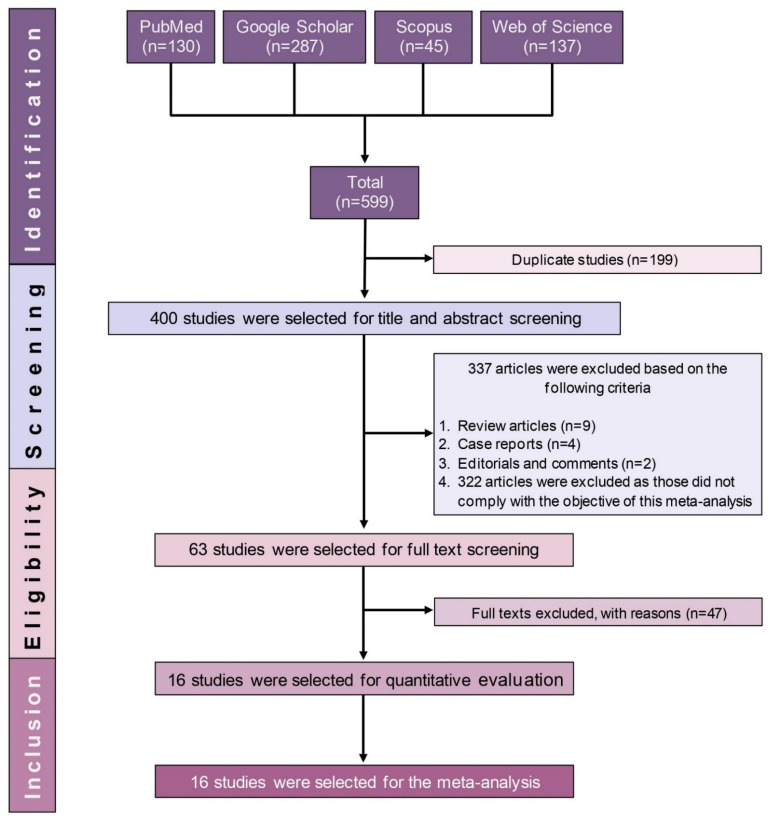
PRISMA flow diagram of study selection.

A flow diagram shows the results of the literature search, and the study selection process is presented in Figure 1. A total of 599 potentially relevant articles were initially identified in the systematic literature search, of which 199 were duplicates, 337 were excluded based on the review of their titles and abstracts, and 63 articles were retained for assessment. After full-text evaluation, only 16 articles addressing the prevalence of drug-resistant TB were included in this SRMA.

### 2.2. Characteristics of the Included Studies

The detailed characteristics of the 16 included studies are summarised in Table 1. The 16 included studies had cross-sectional designs. A total of 1786 Sudanese TB patients were identified and subjected to drug susceptibility testing. The mean age of the patients had a range of 5–90 years. Of the 16 articles, 13 provided data on any drug-resistant TB, 15 provided data on MDR, 13 provided data on isoniazid and 14 on rifampicin, 7 contained information on new TB cases and 8 provided data on previously treated cases.

### 2.3. Quality Assessment and Publication Bias

**Figure 2:**
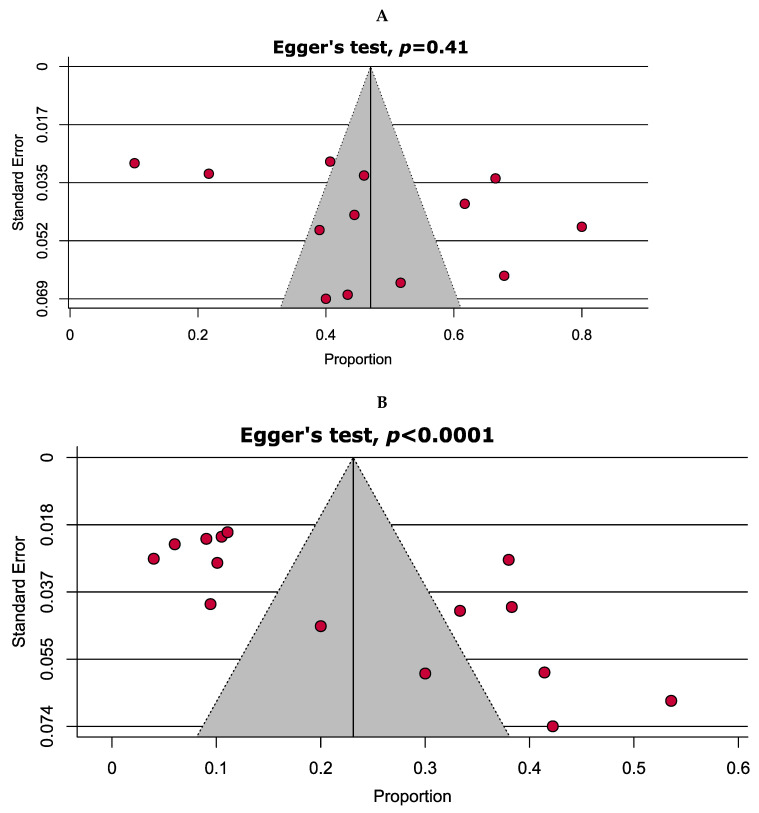
Funnel plots analysing publication bias among studies evaluated (**A**) any drug resistance and (**B**) multidrug resistance.

Quality assessment of the included studies using the JBI critical appraisal checklist for cross-sectional studies is shown in Table S1. Briefly, 11 (68.7%) studies were assessed as having a low risk of bias (high quality), whereas the remaining 5 (31.3%) studies were assessed as having a moderate risk of bias. Assessment of publication bias with funnel plots was possible only in two analyses. Symmetrical and asymmetrical funnel plots (Figure 2) indicated the absence and existence of publication bias, respectively, which were statistically verified through Egger’s test (*p* = 0.41 and <0.0001).

### 2.4. Overall Antibiotic Resistance Patterns 

**Figure 3:**
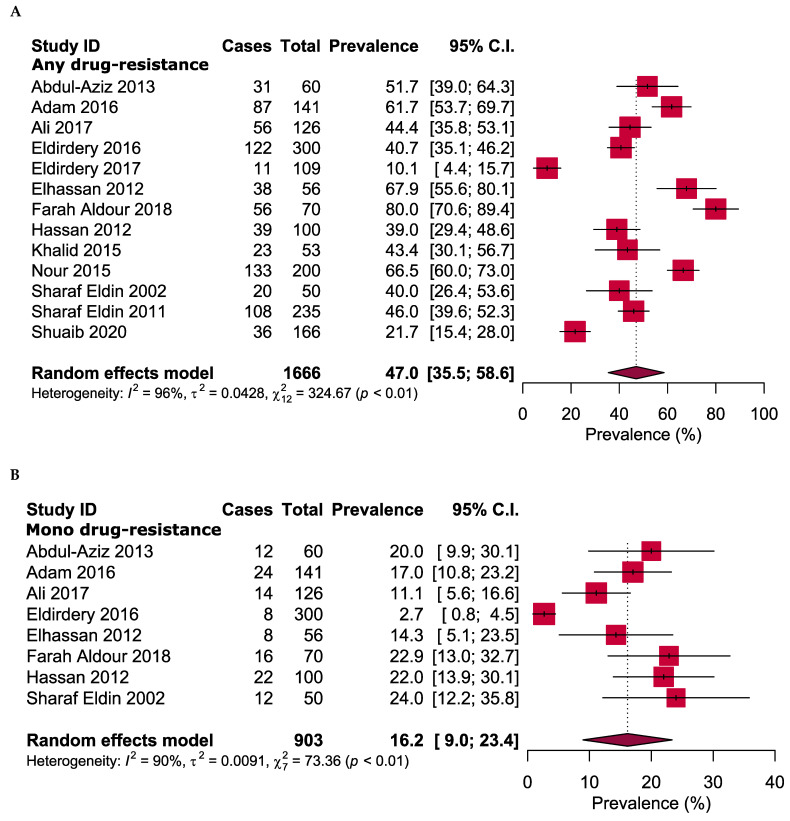

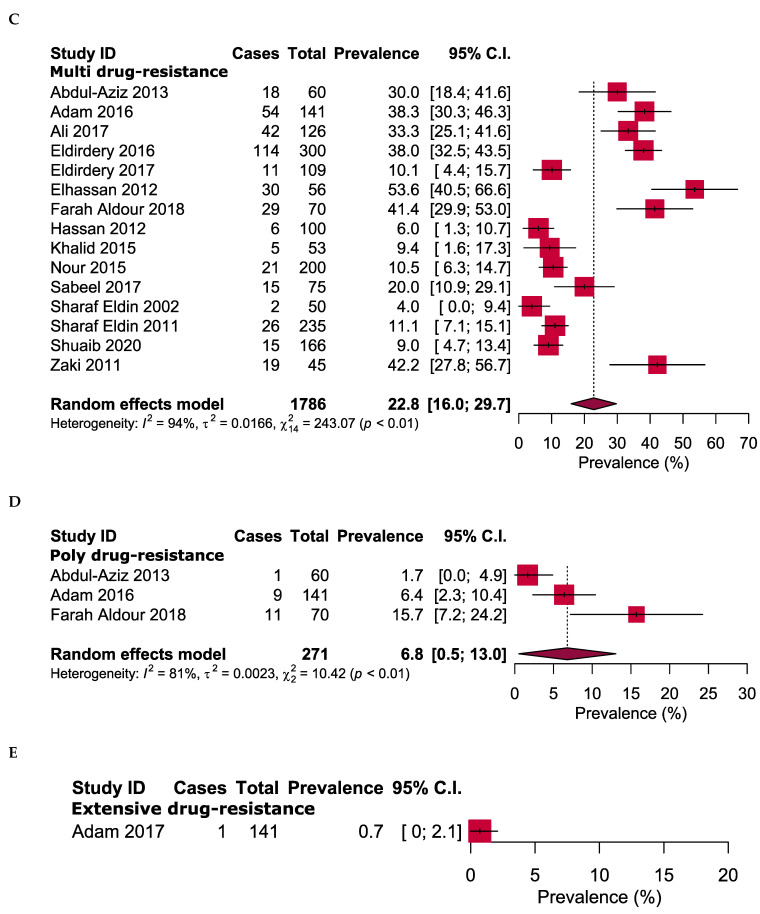
Prevalence of (**A**) any drug resistance, (**B**) mono drug resistance, (**C**) multidrug resistance, (**D**) poly drug resistance and (**E**) extensive drug resistance in pulmonary tuberculosis in Sudan.

Figure 3 shows the overall antibiotic resistance prevalence in the positive cases of TB in Sudan. The meta-analysis revealed that 47.0% (95% CI: 35.5%–58.6%) of all TB cases had resistance to at least one of the tested anti-TB drugs (resistance to any drug). The overall prevalence of mono-resistant TB was 16.2% (95% CI: 9.0%–23.4%), multi drug-resistant TB had 22.8% (95% CI: 16.0%–29.7%) and poly-resistant TB had 6.8% (95% CI: 0.5%–13.0%). Only one XDR isolate was identified, with an estimated prevalence of 0.7% (95% CI: 0%–2.1%).

### 2.5. Resistance to First-Line Anti-TB Drugs

The results of the drug susceptibility tests for any of the five reported first-line anti-TB drugs are detailed in Table 2 and Figure S1. Resistance to isoniazid was the most common, detected in 32.3% (95% CI: 23.6–41.1%) of the resistant strains, followed by resistance to streptomycin (31.7%; 95% CI: 24.6–38.8%). The proportion of patients with rifampicin, ethambutol and pyrazinamide resistance were 29.2%, 15.7% and 10.5%, respectively. In addition, mono resistance for streptomycin (14.0%), isoniazid (2.8%), rifampicin (0.7%), and ethambutol (2.1%) was found (Table 2 and Figure S2).

### 2.6. Resistance to Second-Line Anti-TB Drugs

The resistance profiles of the second-line drug were only reported for two antibiotics, namely ofloxacin and kanamycin, and the corresponding pooled prevalence was estimated. Resistance prevalence to ofloxacin (2.1%, 95% CI: 0–4.5%) and kanamycin (0.7%, 95% CI: 0–2.1%) were notably low (Table 2 and Figure S1).

### 2.7. Drug-Resistance Pattern Based on Treatment History

The patterns of DR-TB according to treatment status (new or previously treated cases) are shown in Table 3. The meta-analysis result revealed that 30.7% and 62.8% of newly diagnosed and previously treated TB patients were resistant to at least one drug (Figures S3–S5). Mono-resistance, MDR and poly-resistance rates were 21.2%, 11.4% and 2.2%, respectively, for the new TB cases and 18.8%, 41.5% and 7.3%, respectively, for the re-treatment cases (Figures S3–S5). Concerning the first-line drugs, resistance was remarkably high among the previously treated patients compared with that among the new cases. Resistance to streptomycin was the most common, with rates of 22.1% (newly treated cases) and 51.1% (re-treatment cases; Table 3 and Figures S3 and S4). Rates of resistance to isoniazid (42.8%) and rifampicin (39.3%) were notably high among re-treatment cases but low in newly diagnosed cases (Table 3 and Figure S4). Notably, resistance to second-line drugs was only reported among re-treatment cases. The prevalence of mono-resistance for first-line drugs ranged from 0.2% (rifampicin) to 13.5% (streptomycin) in new cases and from 1.4% (rifampicin) to 12.2% (streptomycin) for previously treated cases (Figures S4 and S5). 

### 2.8. Time Trend of Anti-TB DR in Sudan

**Figure 4:**
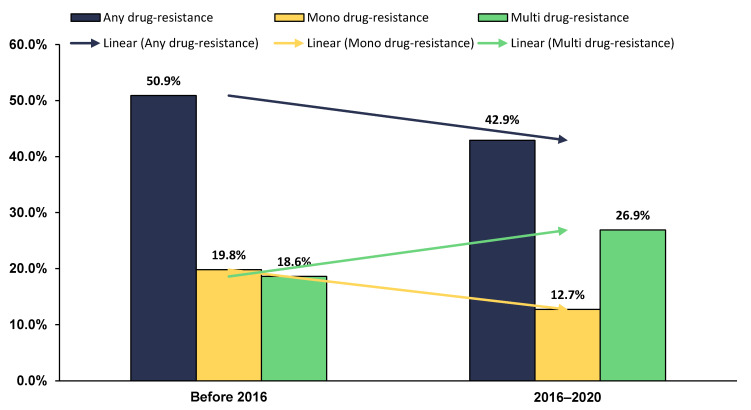
Anti-tuberculosis antibiotic resistance patterns in Sudan.

The assessment of the trend in the prevalence of DR-TB in Sudan during an 18-year timeframe showed clear evidence of declining trend of any drug-resistant (from 50.9% to 42.9%) and mono-resistant TB (from 19.8% to 12.7%). However, the comparative analysis of the nationwide prevalence of MDR-TB over the same period revealed a worsening trend (from 18.6% before 2016 to 26.9% in the next five years) (Figure 4). 

### 2.9. Sensitivity Analyses 

Sensitivity analyses for assessing overall TB antibiotic resistance rates by excluding small studies and studies with low and moderate quality, and by using a fixed-effects model, which showed marginal differences in the re-estimated overall prevalence, and rates ranging from 10.9% lower to 4.8% higher were excluded (Table 4 and Figure S6). Overall, no study has significantly influenced the overall pooled estimate of DR-TB, and thus, all the outcomes are robust and reliable.

## 3. Discussion

Availability of comprehensive data on the prevalence and patterns of DR-TB in endemic areas are extremely essential for designing targeted strategies. To the best of our knowledge, this SRMA is the first to address the prevalence of drug-resistant TB in Sudan. It is worth mentioning that the estimation of DR-TB presented by the TB control programs in Sudan are based on systematic sampling from TB treatment centres or potentially endemic settings using a standardised drug susceptibility test. In contrast, the findings of this SRMA were generated by pooling eligible data on the prevalence of DR-TB reported in 16 published studies. As expected, outcomes were heterogeneous, which was most likely due to the methodological variations between the included studies, sample size and settings. Therefore, the calculated estimates might be confounded by the aforementioned factors and might not truly represent the TB-resistant population in Sudan; nonetheless, it is a first step in raising awareness about Sudan’s potentially alarming TB drug-resistant situation.

The pooled estimate revealed that 47.0% of all TB cases, 30.7% of new cases and 62.8% of previously treated cases from different parts of Sudan had resistance to at least one antibiotic. This suggests that the country may have a high rate of DR-TB, which will likely spread to communities if not appropriately contained. The overall prevalence of any anti-TB-DR (47.0%) identified in the current study was consistent with the finding of similar comprehensive estimates from Bangladesh (resistance to any drug = 45.3%) [31]. However, it was higher than the resistance reported in India (40%) [32] and China (31.1%) [33]. In general, differences in resistance rates among countries might be attributed to the variations in baseline resistance, population density, nature of study participants and many other factors. Of note, the proportion of the overall drug resistance in this study was higher probably because the majority of the included studies were conducted in TB treatment clinics where patients were more likely to have resistant strains. Therefore, the calculated estimates might not accurately represent the Sudanese TB-resistant population. Furthermore, TB patients were recruited from Khartoum in 11 of the 16 included studies, despite the fact that the patients were referred from different regional hospitals. This prevented the synthesis of DR-TB pattern based on the country regions. This raises further concerns about the exact status of the regional prevalence of DR-TB.

According to the literature, the history of previous treatments is one of the most important risk factors associated with DR-TB [34,35]. Previously treated TB patients are more likely to harbour DR-TB strains than new cases [36]. Consistently, this finding was also evidenced by the results of this SRMA, which indicated that almost two-thirds (62.8%) of TB patients with treatment history were resistant to at least one anti-TB drugs compared with only 30.7% of new cases. This observation was similar to the prevalence reported in previously treated patients from Iran (65.6%) [37] and India (58.4%) [38]. Among new cases, the pooled prevalence of any resistance was comparable with the estimate of a previous meta-analysis by Onyedum et al. [39], who found that 32.0% of new TB cases were resistant to at least one drug. In contrast, some studies have reported a lower number of drug-resistant cases among newly diagnosed patients from China (20.1%) and Iran (23%) [37,40]. 

Concerning the first-line drugs, the standardised regimen for anti-TB treatment includes five essential antibiotics: isoniazid, rifampicin, pyrazinamide, ethambutol and streptomycin, according to the Sudan national TB management guideline, 2018 [41]. New and re-treatment TB patients typically receive a standard first-line treatment regimen that consists of two months of isoniazid, rifampicin, pyrazinamide and ethambutol (fixed-dose combinations), followed by four months of isoniazid and rifampicin. In general, high rates of resistance to isoniazid, streptomycin and rifampicin are unfortunate realities in the majority of TB-endemic countries. Such high rates could reflect the frequent, unjustified and inadequate use of antibiotics in general care. In Sudan, the current study revealed that resistance to isoniazid (32.3%) and streptomycin (31.7%) was the most prominent among all patients with TB. Similarly, in many other studies conducted in Bangladesh, Iran and China, resistance to these antibiotics is more common than resistance to other first-line drugs [31,37,42]. Although the resistance to isoniazid among the previously treated patients (42.8%) and newly diagnosed patients (15.7%) was high in the current study, it is still less than that reported from India (38.8% in new cases and 79.5% in previously treated patients) [43]. Regardless of the high rate of isoniazid resistance in many parts of the world, isoniazid remains one of the most effective anti-TB drugs. However, the high resistance rate in this study should not be neglected because isoniazid-resistant precursors might accumulate in the country endemic settings. Consequently, the likelihood of developing MDR-TB could significantly increase if rifampicin resistance increased. Unfortunately, resistance to any rifampicin in the present study was also high among all TB cases (29.2%) and in previously treated patients (39.3%). By contrast, the prevalence was low in new cases (14.8%). This finding is consistent with the SRMA reported in Bangladesh [31], but it is higher than that obtained in studies in Ethiopia and Rwanda [38,44]. The high rate of rifampicin resistance among previously treated patients in this study necessitates implementing an improved monitoring system for all patients undergoing treatment to limit the emergence of more drug-resistant strains.

The overall estimated mono-resistance rate in Sudan was 16.2%, which was higher than the prevalence (14.3%) reported from Bangladesh [31]. The results of this study showed that the rate of mono-resistant isolates among new TB cases was 21.2%, which was higher than that in Iran (17.1%) [37] and in China (10.8%) [40]. Conversely, the prevalence of mono-resistance TB in previously treated patients (18.8%) was close to the prevalence reported in Nigeria (17.0%) [39] but higher than that in Iran (22.0%) [37].

Although rifampicin and isoniazid are major components of antibiotic regimens used in the treatment of TB, the high resistance burden of both drugs has resulted in a relatively high resistance rate of MDR. In Sudan, MDR-TB has not been sufficiently addressed because of the lack of adequate resources for control efforts. Based on this meta-analysis, the MDR-TB prevalence was estimated to be 11.4% in newly diagnosed patients and 41.5% of previously treated patients, with an overall prevalence of 22.8%. This finding is remarkably higher than that of a previous meta-analysis [45], which revealed that 21.07% of the previously treated patients in Ethiopia have MDR-TB. Likewise, the rates of MDR-TB in new and previously treated patients in Sudan are higher than those documented in other studies conducted in Nigeria [39], Ethiopia [46], sub-Saharan Africa [47] and China [40,48]. Consistent with various previous reports, our findings further confirmed the high association of MDR-TB with the history of previous TB treatment.

Nevertheless, an inadequate treatment with insufficient duration and TB infection relapse may significantly contribute to the high prevalence of MDR-TB [49]. Furthermore, several anthropogenic factors, such as poor prescribing practices among medical doctors and poor drug selection, are also associated with acquired resistant TB. It is known that when first-line drugs fail in the treatment of patients with MDR-TB, second-line anti-TB drugs should be used, but they are more toxic, less effective and more expensive than first-line drugs. Thus far, only a few studies have reported the resistance to second-line drugs in Sudan. In the present study, resistance was only observed in ofloxacin (2.1%), kanamycin (0.7%) and para-aminosalicylic acid (22.7%). The resistance frequency of the injectable second-line drug kanamycin was lower than that found in Zimbabwe (5%) [50] and China (16.7%) [51]. Similarly, the low resistance rate to ofloxacin in this study was consistent with the previous finding from Georgia in which resistance to ofloxacin was found to be 2.2%; however, resistance to kanamycin in Georgia was slightly high compared to this study [52]. 

With the improper usage of second-line drugs, XDR-TB has developed and subsequently emerged. These strains are highly resistant and often associated with high morbidity, treatment failure and mortality. Thus, the growing trend of resistance to second-line anti-TB drugs and XDR-TB is alarming. Fortunately, this meta-analysis captured only one study in Sudan reporting a very low proportion of XDR-TB (0.7%). A similar rate of XDR-TB was reported in Pakistan [53]. However, the findings of one study may not provide sufficient detailed information and should be confirmed with additional testing of TB isolates. Therefore, further studies should also be performed to explore the burden of XDR-TB in Sudan.

The prevalence of DR-TB in Sudan over an 18-year period revealed clear evidence of a declining trend in any drug-resistant TB. This finding is inconsistent with the remarkable increase in any drug-resistant TB, which was recently documented in Bangladesh between 2011 and 2018, when compared to 1999 and 2010 [31]. On the other hand, the prevalence of MDR-TB was found to be higher (26.9%) in the most recent studies (2016–2020), as compared to 18.6% for studies conducted before 2016. Such an increasing trend has been similarly reported in various recent studies conducted in Bangladesh [31] and India [32]. On the other hand, a declining trend in the MDR-TB prevalence was observed sub-Saharan Africa during the period between 1995 and 2015 [47]. Perhaps the increasing trend of MDR-TB reported in this SRMA may not necessarily reflect the situation of MDR-TB on a national scale, but it does partially reveal some defects in the current TB control program.

As a direct implication of this study for TB control in Sudan, several influencing factors for the development of DR-TB and MDR-TB in Sudan should be considered immediately in order to prevent the accelerating spread of DR-TB. First, the key issue that must be addressed is the DR-TB detection system [54], given that the National Reference TB Laboratory in Khartoum is the only reference laboratory in the country [55]. The establishment of a rapid identification system and the strengthening of the capacity of TB reference and zonal laboratories would allow for the proper detection, treatment and management of drug-susceptible TB, thereby preventing the development of DR-TB [56].

Second, implementing effective control measures in TB treatment clinics and hospitals has a high potential for protecting both other patients and healthcare workers. In this regard, control measures consisting of administrative, environmental and personal respiratory protective equipment usage would be an adequate health system response. On the other hand, creative strategies to limit transmission inside community hotspots must be considered [57]. At the community level, it is important to screen household contacts, symptomatic individuals and high-risk groups, particularly children and immunocompromised patients, followed by a supervised treatment, which plan may play a crucial role in reducing the risk of transmission [56]. Furthermore, engaging the entire healthcare system in TB-related activities is also important, besides empowering individuals and communities to support a TB control plan and reduce TB-related stigma [58].

Third, the rational use of anti-TB drugs has been a problem in developing countries; thus, the availability of therapeutic procedures within the framework of internationally recognised standardised treatment regimens, particularly those recommended by the WHO, might be the basis of preventing and containing the further spread of DR-TB [59,60].

Finally, it is clear that the majority of the studies included in this review were limited by cost and accessibility of collecting samples from endemic areas rather than TB treatment clinics. Despite the alarming results, in most of the included studies DR-TB rates were obtained from a smaller sample size. Therefore, expanded surveillance as well as additional studies with a large and systematic sample collection covering various geographical regions across the country are highly recommended [61].

A key strength of this SRMA is that it is the first to provide a comprehensive estimation of drug-resistant TB in Sudan. However, it had several limitations. First, the included studies did not encompass all the states of the country, so the estimated prevalence might not fully represent the magnitude of drug-resistant TB in Sudan. Second, substantial heterogeneity was observed in the included studies, although this observation is common in meta-analyses on estimating prevalence [62,63,64]. Third, all of the included studies were hospital-based studies and none were community-based studies, making the results more susceptible to potential selection bias. Finally, the potential effect of gender, age, socioeconomic status and lifestyle of the included patients on the prevalence of DR-TB could not be analysed because of the unavailability of data in many of the included studies. 

## 4. Methods

### 4.1. Reporting Guideline and Protocol Registration

This study was conducted in compliance with the guidelines of the updated Preferred Reporting Items for Systematic Reviews and Meta-analyses (PRISMA) [65]. The protocol of this study was registered on the International Prospective Register of Systematic Reviews (PROSPERO) database (registration number: CRD42021249885).

### 4.2. Search Strategies

A comprehensive literature search was conducted to find studies on DR-TB prevalence in Sudan that were available in the PubMed, Scopus, Web of Science and Google Scholar databases. In addition, the reference lists of the included articles were also checked for the identification of studies that we missed through the search strategies. The detailed search strategy for all databases is presented in Table S2.

### 4.3. Selection Criteria

Studies that reported or provided adequate data for calculating the prevalence of antibiotic resistance in all, newly and/or previously treated TB patients from Sudan, regardless of age, gender or language restrictions, and regardless of the period in which the studies were conducted or published, were considered eligible for inclusion in this SRMA. By contrast, studies that reported extrapulmonary TB or considered only TB cases co-infected with HIV infection, thesis, review articles, case reports, case studies and studies with abstracts only were excluded.

### 4.4. Data Management and Study Selection

All identified studies were retrieved and managed using EndNote (Clarivate Analytics, Boston, MA, USA). Duplicates were removed, and the remaining papers were evaluated extensively based on their titles and abstracts. Full texts were further assessed for the identification of eligible studies. Two authors (K.H. and M.M.) independently evaluated the studies’ eligibility using predetermined criteria. Discrepancies were resolved by discussing with the third author (M.A.I.) until a consensus was reached.

### 4.5. Operational Definitions

The following definitions were adopted in compliance with the DR-TB guidelines that were used in this meta-analysis [66]. First-line drugs: rifampicin, isoniazid ethambutol, pyrazinamide and streptomycin. Second-line drugs: ofloxacin, moxifloxacin, levofloxacin, ciprofloxacin, kanamycin, capreomycin, amikacin, ethionamide, prothionamide, cycloserine and para-aminosalicylic acid. Mono resistance: resistance to only one first-line anti-TB drug. Poly resistance: other than isoniazid and rifampicin, resistance to more than one first-line anti-TB drug. Multidrug resistance (MDR): resistance to at least both isoniazid and rifampicin. Any drug resistance was defined as resistance to any drug regardless to mono resistance or MDR. Extensive drug resistance (XDR): multidrug resistance, fluoroquinolone resistance and resistance to at least one of the three second-line injectable drugs.

### 4.6. Data Extrction

Two reviewers (K.H. and M.M.) independently extracted relevant data or information from the included studies using a standardised data extraction form. To minimise errors and ensure consistency, a third author (M.I.I.) subsequently double-checked the extracted data or information. From each eligible study, the following data were extracted: last name of the first author, year of publication, study design, study enrolment time, region/province, distributions of gender and age in the study population, number of TB-positive patients, drug susceptibility test (DST) and the prevalence of drug resistance (any, mono, multi and poly resistant).

### 4.7. Quality Assessment

The quality of the included studies was independently evaluated by two of the authors (K.H. and M.A.I.) using the Joana Brigg’s Institute (JBI) critical assessment checklist for cross-sectional studies [67]. The results of the two authors’ assessment were then compared, and notable discrepancies were identified. Consequently, any disagreement was resolved by consensus. Studies with scores of >70% reporting ‘yes’ were considered to have a low risk of bias (high quality). Studies with scores of 50%–70% were considered to have a moderate risk of bias (moderate quality). Finally, studies with scores of <50% reporting ‘yes’ were considered to have a high risk of bias [68]. 

### 4.8. Data Analyses 

Meta-analysis was carried out using metaprop codes in the meta (version 4.15-1) and metafor (version 2.4-0) packages of R (version 3.6.3) in RStudio (version 1.3.1093). The pooled estimates of resistance to any drugs and mono resistance, MDR, poly resistance and XDR and 95% confidence intervals (CIs) were calculated using the REML method for the random-effects models. Heterogeneity between studies was measured using *I*^2^ statistics and Cochran’s Q-test. The *I*^2^ statistic of >75% and a significance level of <0.05 were interpreted as evidence of substantial heterogeneity. The presence of publication bias was checked by visually inspecting the funnel plot tested for significance with Egger’s test. However, the insufficient number of included studies (less than 10) rendered the use of the Funnel plot technique in assessing publication bias unnecessary. 

### 4.9. Subgroup and Sensitivity Analyses

For exploring the potential sources of heterogeneity, a subgroup analysis of DR-TB prevalence was carried out according to specific first- and second-line drugs and resistance patterns in new cases and previously treated cases. The robustness of the pooled estimates was validated through sensitivity analysis, which was conducted according to the following strategies: exclusion of small studies (*n* < 100), exclusion of low- and moderate-quality studies and uses of a fixed-effects model.

## 5. Conclusions

The pooled estimates demonstrated a relatively high burden of DR-TB in Sudan posing a new challenge to public health. Our results suggested that drug susceptibility test should be initiated and scaled up quickly in order to identify resistant strains rapidly. In addition, robust national surveillance systems should be established for the effective treatment, prevention and continuous monitoring of drug-resistant TB in Sudan.

## Figures and Tables

**Table 1:** Major characteristics of the included studies.

No.	Study ID [References]	Enrolment Time	Study Area	Sample Size	Gender		Age in Years (Range)	TB-Positive Cases	Drug Susceptibility Tests	Tested Antibiotic
					M	F				
1	Abdul-Aziz 2013 [15]	2011	Kassala	90	54	36	14–65	60	LJ proportion method and SSCP	STM, RIF, INH, EMB and ETH
2	Adam 2016 [16]	2009–2010	Khartoum	239	175	64	13–75	141	LJ proportion method	RIF, INH, EMB and STM
3	Adam 2017 [17]	2009–2010	Khartoum	239	175	64	13–75	141	LJ proportion method and by Hain GenoType MTBDRsl Assay	RIF, INH, STM, EMB, KAN, CAP, OFX and AMK
4	Ali 2017 [18]	2011–2015	Khartoum	126	85	41	16–30	126	Conventional DST, LPA and GeneXpert assay	RIF and INH
5	Eldirdery 2016 [19]	NR	Khartoum	300	NR	NR	NR	300	LJ Proportion method and LPA	INH, RIF, STM and EMB
6	Eldirdery 2017 [20]	2011–2012	Kassala and Geddarif	109	64	45	13–80	109	LJ Proportion method and LPA	INH, RIF, STM and EMB
7	Elhassan 2012 [21]	NR	Khartoum	130	82	48	12–67	56	LJ proportion method and PCR	INH and RIF
8	Farah Aldour 2018 [22]	2015	Omdurman	70	NR	NR	10–80	70	Multiplex PCR	RIF, INH and PZA
9	Hassan 2012 [23]	2006–2007	Port Sudan	100	68	32	≥18	100	LJ proportion method and MAS-PCR	RIF, INH, STM, EMB and PZA
10	Khalid 2015 [24]	2007–2009	Kassala	53	NR	NR	NR	53	LJ proportion method	INH, RIF, STM and EMB
11	Nour 2015 [25]	NR	Khartoum	NR	NR	NR	5–70	200	LJ proportion method	INH, RIF, STM and EMB
12	Sabeel 2017 [26]	NR	Khartoum	100	NR	NR	NR	75	LJ proportion and PCR	INH, RIF, STM and EMB
13	Sharaf Eldin 2002 [27]	1998–1999	Khartoum	105	NR	NR	NR	50	PCR-based dot-blot method	INH, RIF, STM, PZA and EMB
14	Sharaf Eldin 2011 [28]	2005	Khartoum and Port Sudan	235	175	60	26–45	235	LJ proportion method	INH, RIF, STM and EMB
15	Shuaib 2020 [29]	2014–2016	Kassala, Port Sudan, and El-Gadarif	383	245	138	25–45	166	Phenotypic and genotypic DST	RIF, INH, EMB, STM and PZA
16	Zaki 2011 [30]	2007–2007	Khartoum	111	83	28	NR	45	LJ proportion method	RIF, INH, STM and EMB

RIF: rifampicin, INH: isoniazid, STM: streptomycin, EMB: ethambutol, PAS: para-aminosalicylic acid, KAN: kanamycin, CAP: capreomycin, OFX: ofloxacin, AMK: amikacin, SSCP: single-strand DNA conformation polymorphism analysis, MAS-PCR: multiplex allele specific polymerase chain reaction, NI: not included and NR: not reported.

**Table 2:** Any and mono anti-tuberculosis DR patterns in Sudan.

Drug-Resistance Patterns		Antibiotics	Number of Analysed Studies	Total Number of Tuberculosis Patients	Prevalence of Antibiotic Resistance [95% CIs] (%)	Heterogeneity	
						*I* ^2^	*p*-Value
**Any DR**	**First-line drugs**	Streptomycin	10	1125	31.7 [24.6–38.8]	86%	<0.0001
		Isoniazid	13	1624	32.3 [23.6–41.1]	94%	<0.0001
		Rifampicin	14	1677	29.2 [21.4–36.9]	94%	<0.0001
		Ethambutol	9	1072	15.7 [8.0–23.4]	95%	<0.0001
		Pyrazinamide	3	336	10.5 [2.8–18.1]	97%	<0.0001
	**Second-line drugs**	Kanamycin	1	141	0.7 [0.0–2.1]	NA	NA
		Ofloxacin	1	141	2.1 [0.0–4.5]	NA	NA
**Mono DR**	**First-line drugs**	Streptomycin	4	351	14.0 [9.9–18.1]	20%	0.29
		Isoniazid	7	833	2.8 [1.2–4.5]	48%	0.07
		Rifampicin	7	833	0.7 [0.0–1.5]	16%	0.38
		Ethambutol	3	301	2.1 [0.5–3.7]	0%	0.40

CI: confidence interval; DR: drug resistance; NA: not applicable.

**Table 3:** Anti-tuberculosis DR patterns in newly diagnosed and previously diagnosed tuberculosis patients from Sudan.

Drug-Resistance Patterns		Antibiotics	Number of Analysed Studies	Total Number of Tuberculosis Patients	Prevalence of Antibiotic Resistance [95% CIs] (%)	Heterogeneity		
						*I* ^2^	*p*-Value	
**Newly diagnosed tuberculosis patients**								
**Any DR**	**First-line drugs**	Streptomycin	4	321	22.1 [10.7–33.6]	80%	0.001	
		Isoniazid	3	310	15.7 [7.3–24.1]	74%	0.02	
		Rifampicin	4	321	14.8 [7.5–22.1]	65%	0.03	
		Ethambutol	3	310	7.9 [3.8–12.1]	38%	0.19	
		Pyrazinamide	1	100	1.0 [0.0–3.0]	NA	NA	
**Mono DR**	**First-line drugs**	Streptomycin	2	146	13.5 [4.4–22.6]	63%	0.10	
		Isoniazid	2	146	1.5 [0.0–3.8]	11%	0.29	
		Rifampicin	2	146	0.2 [0.0–1.5]	0%	0.45	
		Ethambutol	2	146	3.3 [0.4–6.2]	0%	0.69	
**Previously treated tuberculosis patients**								
**Any DR**	**First-line drugs**	Streptomycin	3	226	51.1 [26.1–76.1]	92%	<0.0001	
		Isoniazid	4	296	42.8 [37.2–48.4]	0%	0.54	
		Rifampicin	4	296	39.3 [33.4–45.2]	8%	0.35	
		Ethambutol	3	226	39.4 [13.0–65.8]	93%	<0.0001	
		Pyrazinamide	1	70	47.1 [35.4–58.8]	NA	NA	
	**Second-line drugs**	Kanamycin	1	141	0.7 [0.0–2.1]	NA	NA	
		Ofloxacin	1	141	2.1 [0.0–4.5]	NA	NA	
**Mono DR**	**First-line drugs**	Streptomycin	2	155	12.2 [7.1–17.4]	0%	0.81	
		Isoniazid	2	155	2.0 [0.0–4.3]	0%	0.80	
		Rifampicin	2	155	1.4 [0.0–3.4]	0%	0.68	
		Ethambutol	2	155	1.5 [0.0–3.5]	0%	0.41	

CI: confidence interval; DR: drug resistance; NA: not applicable.

**Table 4:** Sensitivity analyses.

Strategies of Sensitivity Analyses	Prevalence of Antibiotic Resistance [95% CIs] (%)	Difference of Pooled Prevalence Compared to the Main Result	Number of Studies Analysed	Total Number of TB Patients	Heterogeneity	
					*I* ^2^	*p*-Value
**Any drug resistance**						
Excluding small studies (*n* < 100)	41.2 [27.3–55.1]	6.3% lower	8	1377	97%	<0.0001
Excluding low- and moderate-quality studies	44.9 [31.6–58.1]	2.6% lower	8	1127	96%	<0.0001
Using a fixed-effects model	42.4 [40.2–44.6]	5.1% lower	13	1666	96%	<0.0001
**Mono drug resistance**						
Excluding small studies (*n* < 100)	12.7 [3.5–21.9]	4.8% lower	4	667	93%	<0.0001
Excluding low- and moderate-quality studies	14.0 [6.4–21.6]	3.5% lower	6	783	91%	<0.0001
Using a fixed-effects model	6.6 [5.0–8.1]	10.9% lower	8	903	90%	<0.0001
**Multidrug resistance**						
Excluding small studies (*n* < 100)	19.2 [10.7–27.7]	3.0% lower	8	1377	95%	<0.0001
Excluding low- and moderate-quality studies	27.6 [17.9–37.2]	4.8% higher	10	1247	95%	<0.0001
Using a fixed-effects model	15.7 [14.1–17.3]	7.1% lower	15	1786	94%	<0.0001
**Poly drug resistance**						
Excluding small studies (*n* < 100)	6.4 [2.3–10.4]	0.4% lower	1	141	NA	NA
Excluding low- and moderate-quality studies	3.9 [0.0–8.5]	2.9% lower	2	201	69%	0.70
Using a fixed-effects model	4.5 [2.1–6.9]	2.3% lower	3	271	81%	0.005
**Extensive drug resistance**						
Excluding small studies (*n* < 100)	0.7 [0.0–2.1]	No change	1	141	NA	NA
Excluding low- and moderate-quality studies	0.7 [0.0–2.1]	No change	1	141	NA	NA
Using a fixed-effects model	0.7 [0.0–2.1]	No change	1	141	NA	NA

CIs: confidence intervals, NA: not applicable.

## Data Availability

The data presented in this study are available within the article and Supplementary Materials.

## Supplementary Materials

The following are available online at https://www.mdpi.com/article/10.3390/antibiotics10080932/s1, Figure S1: Any resistance to first- and second-line anti-TB drugs: (A) streptomycin, (B) isoniazid, (C) rifampicin, (D) ethambutol, (E) pyrazinamide, (F) kanamycin, (G) ofloxacin and (H) para-aminosalicylic acid, Figure S2: Mono resistance to anti-TB drugs: (A) streptomycin, (B) isoniazid, (C) rifampicin and (D) ethambutol, Figure S3: Any resistance to anti-TB drugs in newly diagnosed patients: (A) streptomycin (B) isoniazid, (C) rifampicin, (D) ethambutol and (E) pyrazinamide, and any resistance to anti-TB drugs in previously treated patients: (F) streptomycin, (G) isoniazid, (H) rifampicin, (I) kanamycin, (J) ofloxacin and (K) pyrazinamide, Figure S4: Mono resistance to anti-TB drugs in newly diagnosed patients: (A) streptomycin, (B) isoniazid, (C) rifampicin and (D) ethambutol, and mono resistance to anti-TB drugs in previously treated patients: (E) streptomycin, (F) isoniazid, (G) rifampicin and (H) ethambutol, Figure S5: Overall drug resistance (DR) in newly diagnosed and previously treated TB patients. (A) Any DR in newly diagnosed patients, (B) any DR in previously treated patients, (C) mono DR in newly diagnosed patients, (D) mono DR in previously treated patients, (E) multi DR in newly diagnosed patients, (F) multi DR in previously treated patients, (G) poly DR in newly diagnosed patients and (H) poly DR in previously treated patients, Figure S6: Sensitivity analyses: excluding small studies (*n* < 100) from A to E; excluding moderate-quality studies from F to J and using a fixed-effects model from K to O, Table S1: Quality assessment of the included cross-sectional studies, Table S2: Search strategy.
