# Supplementary material for: Prevalence of Drug-Resistant Tuberculosis in Sudan: A Systematic Review and Meta-Analysis
Source: Antibiotics (Basel). 2021 Jul 31;10(8):932. doi: 10.3390/antibiotics10080932 (PMC8388945; doi:10.3390/antibiotics10080932)
Supplement: Supplementary file 1 [file antibiotics-10-00932-s001.zip › antibiotics-1287874-supplementary.pdf]

A

Study ID	Cases	Total	Prevalence	95% C.I.
**Any streptomycin-resistance**				
Abdul-Aziz 2013	11	60	18.3	[8.5; 28.1]
Adam 2016	58	141	41.1	[33.0; 49.3]
Hassan 2012	34	100	34.0	[24.7; 43.3]
Khalid 2015	11	53	20.8	[9.8; 31.7]
Nour 2015	43	200	21.5	[15.8; 27.2]
Sabeel 2017	42	75	56.0	[44.8; 67.2]
Sharaf Eldin 2002	15	50	30.0	[17.3; 42.7]
Sharaf Eldin 2011	76	235	32.3	[26.4; 38.3]
Shuaib 2020	33	166	19.9	[13.8; 26.0]
Zaki 2011	22	45	48.9	[34.3; 63.5]
**Random effects model**		**1125**	**31.7**	**[24.6; 38.8]**

Heterogeneity: I^2 = 86%, τ^2 = 0.0107, χ^2_9 = 62.31 (p < 0.01)

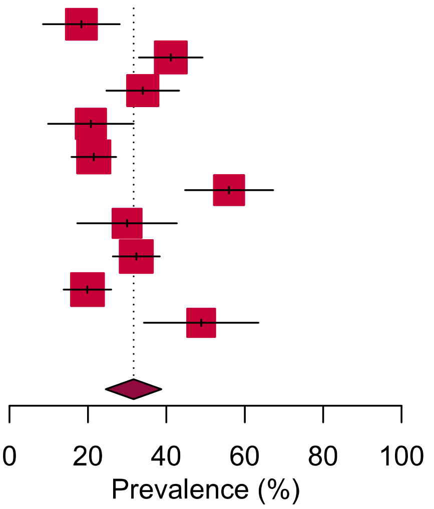

B

Study ID	Cases	Total	Prevalence	95% C.I.
**Any isoniazid-resistance**				
Abdul-Aziz 2013	21	60	35.0	[22.9; 47.1]
Adam 2016	65	141	46.1	[37.9; 54.3]
Ali 2017	52	126	41.3	[32.7; 49.9]
Eldirdery 2016	121	300	40.3	[34.8; 45.9]
Elhassan 2012	36	56	64.3	[51.7; 76.8]
Farah Aldour 2018	29	70	41.4	[29.9; 53.0]
Hassan 2012	13	100	13.0	[6.4; 19.6]
Nour 2015	35	200	17.5	[12.2; 22.8]
Sabeel 2017	21	75	28.0	[17.8; 38.2]
Sharaf Eldin 2002	6	50	12.0	[3.0; 21.0]
Sharaf Eldin 2011	43	235	18.3	[13.4; 23.2]
Shuaib 2020	17	166	10.2	[5.6; 14.9]
Zaki 2011	28	45	62.2	[48.1; 76.4]
**Random effects model**		**1624**	**32.3**	**[23.6; 41.1]**

Heterogeneity: I^2 = 94%, τ^2 = 0.0238, χ^2_{12} = 218.18 (p < 0.01)

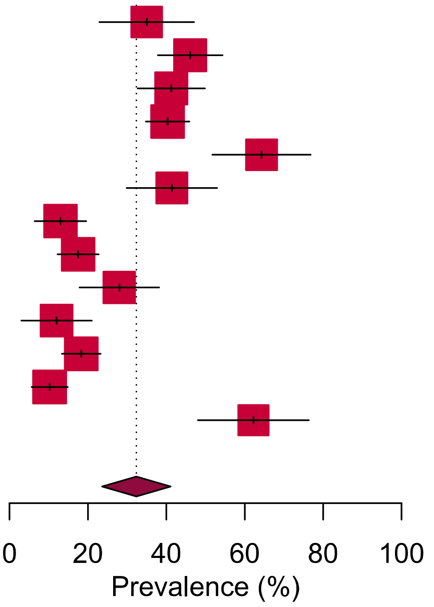

C

Study ID	Cases	Total	Prevalence	95% C.I.
**Any rifampicin-resistance**				
Abdul-Aziz 2013	19	60	31.7	[19.9; 43.4]
Adam 2016	57	141	40.4	[32.3; 48.5]
Ali 2017	46	126	36.5	[28.1; 44.9]
Eldirdery 2016	115	300	38.3	[32.8; 43.8]
Elhassan 2012	32	56	57.1	[44.2; 70.1]
Farah Aldour 2018	29	70	41.4	[29.9; 53.0]
Hassan 2012	8	100	8.0	[2.7; 13.3]
Khalid 2015	13	53	24.5	[12.9; 36.1]
Nour 2015	31	200	15.5	[10.5; 20.5]
Sabeel 2017	22	75	29.3	[19.0; 39.6]
Sharaf Eldin 2002	4	50	8.0	[0.5; 15.5]
Sharaf Eldin 2011	53	235	22.6	[17.2; 27.9]
Shuaib 2020	17	166	10.2	[5.6; 14.9]
Zaki 2011	25	45	55.6	[41.0; 70.1]
**Random effects model**		**1677**	**29.2**	**[21.4; 36.9]**

Heterogeneity: $I^2 = 94\%$, $\tau^2 = 0.0196$, $\chi^2_{13} = 200.59$ ($p < 0.01$)

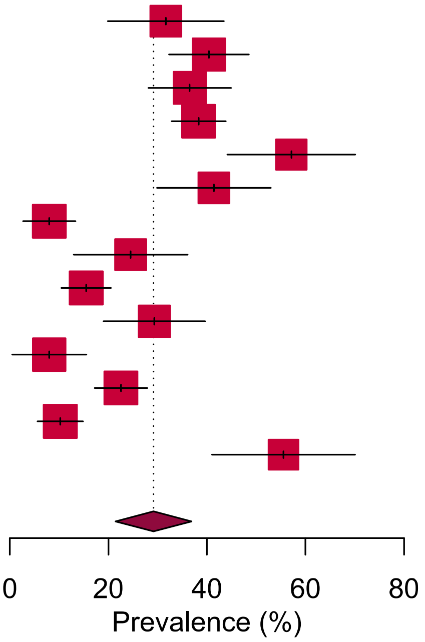

D

Study ID	Cases	Total	Prevalence	95% C.I.
**Any ethambutol-resistance**				
Abdul-Aziz 2013	8	60	13.3	[4.7; 21.9]
Adam 2016	37	141	26.2	[19.0; 33.5]
Hassan 2012	12	100	12.0	[5.6; 18.4]
Nour 2015	24	200	12.0	[7.5; 16.5]
Sabeel 2017	15	75	20.0	[10.9; 29.1]
Sharaf Eldin 2002	2	50	4.0	[0.0; 9.4]
Sharaf Eldin 2011	53	235	22.6	[17.2; 27.9]
Shuaib 2020	1	166	0.6	[0.0; 1.8]
Zaki 2011	17	45	37.8	[23.6; 51.9]
**Random effects model**		**1072**	**15.7**	**[8.0; 23.4]**

Heterogeneity: $I^2 = 95\%$, $\tau^2 = 0.0125$, $\chi^2_8 = 170.52$ ($p < 0.01$)

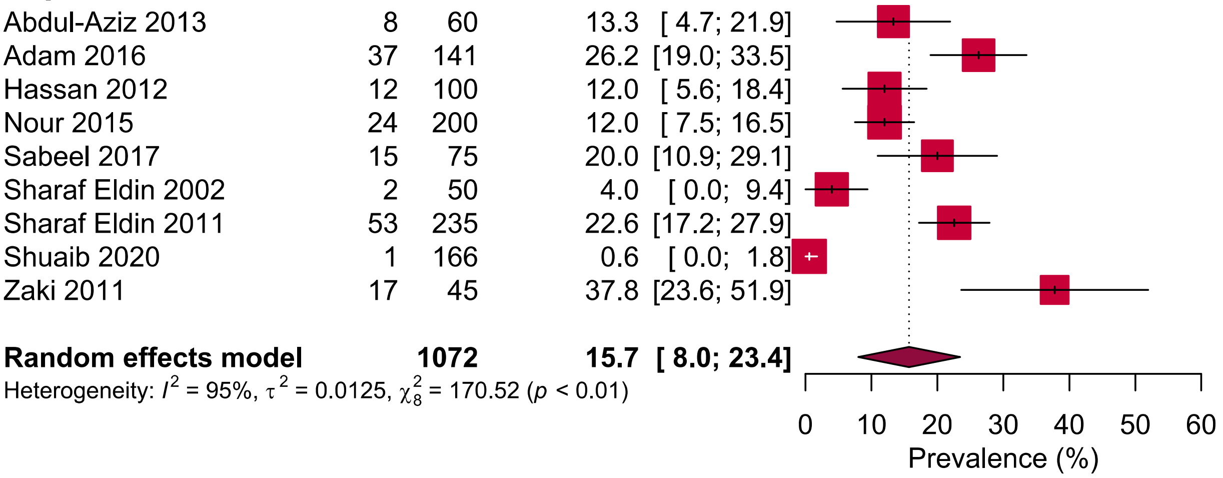

E

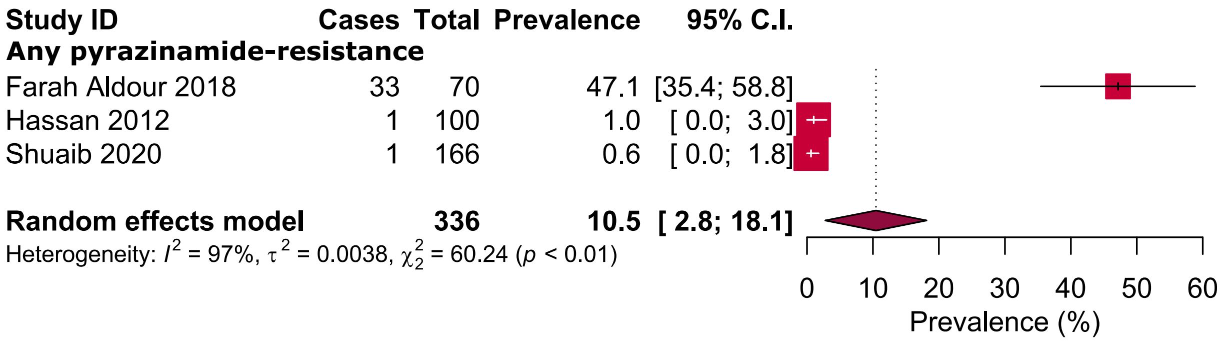

F

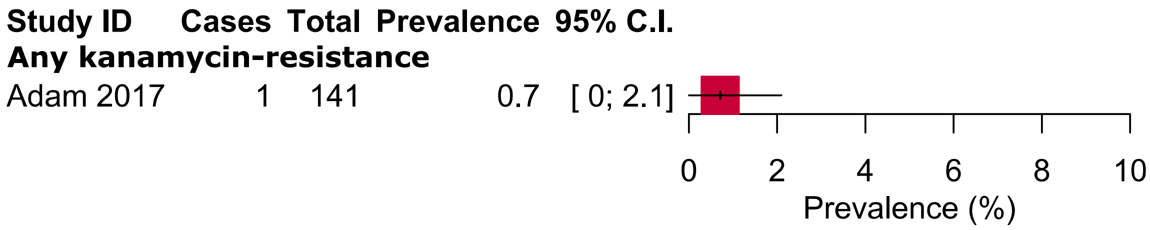

G

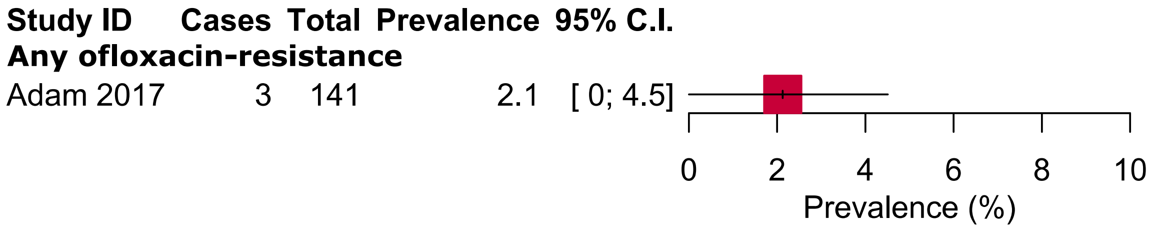

Figure S1. Any resistance to first- and second-line anti-TB drugs: A) streptomycin, B) isoniazid, C) rifampicin, D) ethambutol, E) pyrazinamide, F) kanamycin, and G) ofloxacin.

A

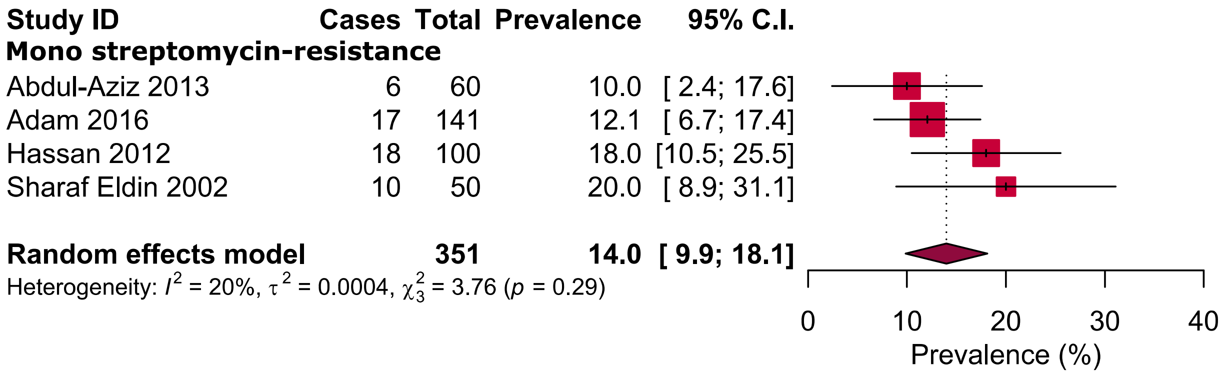
Study ID Cases Total Prevalence 95% C.I.
Mono streptomycin-resistance
Abdul-Aziz 2013 6 60 10.0 [2.4; 17.6]
Adam 2016 17 141 12.1 [6.7; 17.4]
Hassan 2012 18 100 18.0 [10.5; 25.5]
Sharaf Eldin 2002 10 50 20.0 [8.9; 31.1]
Random effects model 351 14.0 [9.9; 18.1]
Heterogeneity: I^2 = 20%, τ^2 = 0.0004, χ^2_3 = 3.76 (p = 0.29)
0 10 20 30 40
Prevalence (%)

B

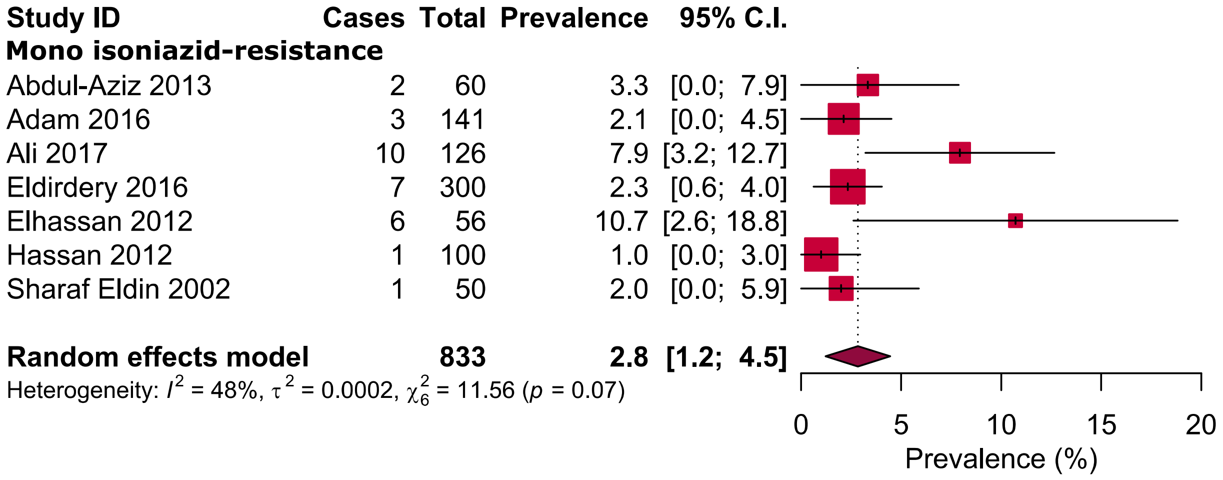
Study ID Cases Total Prevalence 95% C.I.
Mono isoniazid-resistance
Abdul-Aziz 2013 2 60 3.3 [0.0; 7.9]
Adam 2016 3 141 2.1 [0.0; 4.5]
Ali 2017 10 126 7.9 [3.2; 12.7]
Eldirdery 2016 7 300 2.3 [0.6; 4.0]
Elhassan 2012 6 56 10.7 [2.6; 18.8]
Hassan 2012 1 100 1.0 [0.0; 3.0]
Sharaf Eldin 2002 1 50 2.0 [0.0; 5.9]
Random effects model 833 2.8 [1.2; 4.5]
Heterogeneity: I^2 = 48%, τ^2 = 0.0002, χ^2_6 = 11.56 (p = 0.07)
0 5 10 15 20
Prevalence (%)

C

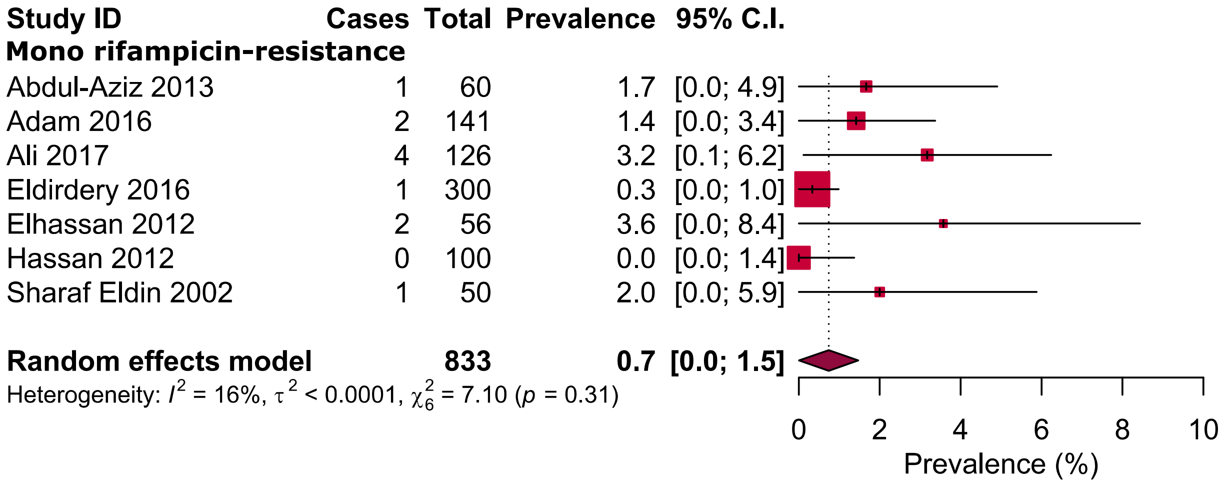
Study ID Cases Total Prevalence 95% C.I.
Mono rifampicin-resistance
Abdul-Aziz 2013 1 60 1.7 [0.0; 4.9]
Adam 2016 2 141 1.4 [0.0; 3.4]
Ali 2017 4 126 3.2 [0.1; 6.2]
Eldirdery 2016 1 300 0.3 [0.0; 1.0]
Elhassan 2012 2 56 3.6 [0.0; 8.4]
Hassan 2012 0 100 0.0 [0.0; 1.4]
Sharaf Eldin 2002 1 50 2.0 [0.0; 5.9]
Random effects model 833 0.7 [0.0; 1.5]
Heterogeneity: I^2 = 16%, τ^2 < 0.0001, χ^2_6 = 7.10 (p = 0.31)
0 2 4 6 8 10
Prevalence (%)

D

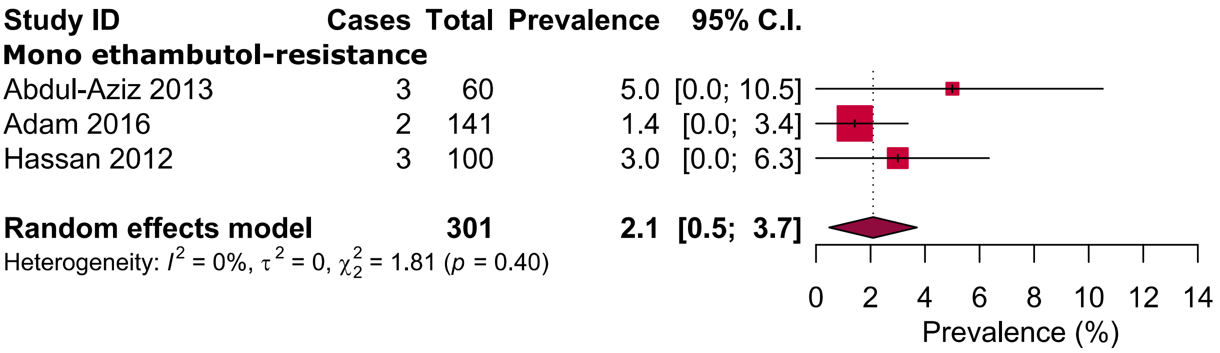

Figure S2. Mono resistance to anti-TB drugs: A) streptomycin, B) isoniazid, C) rifampicin, and D) ethambutol.

A

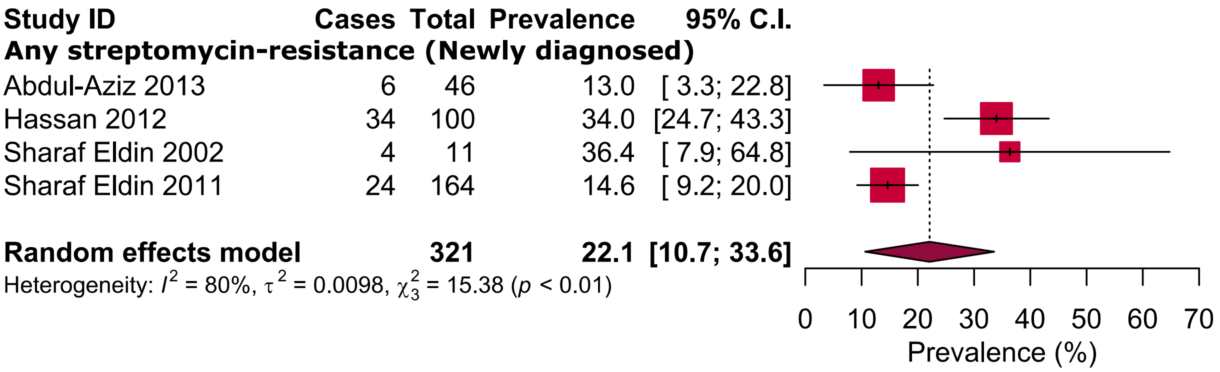

B

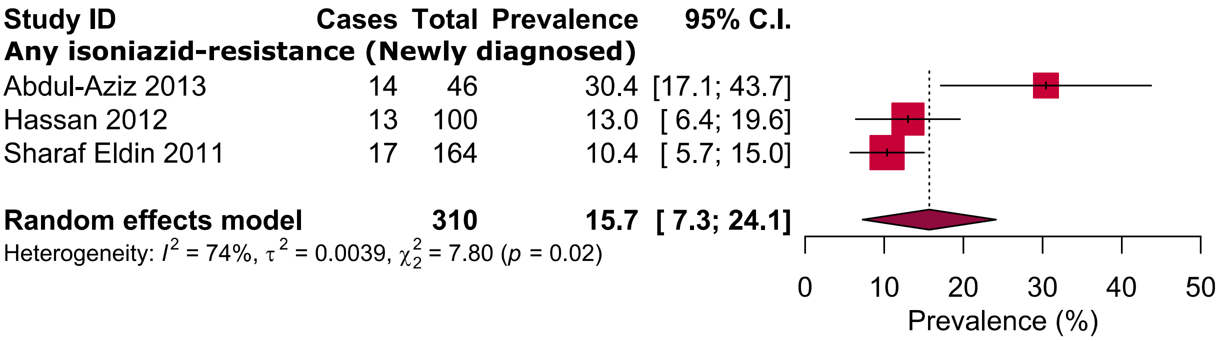

C

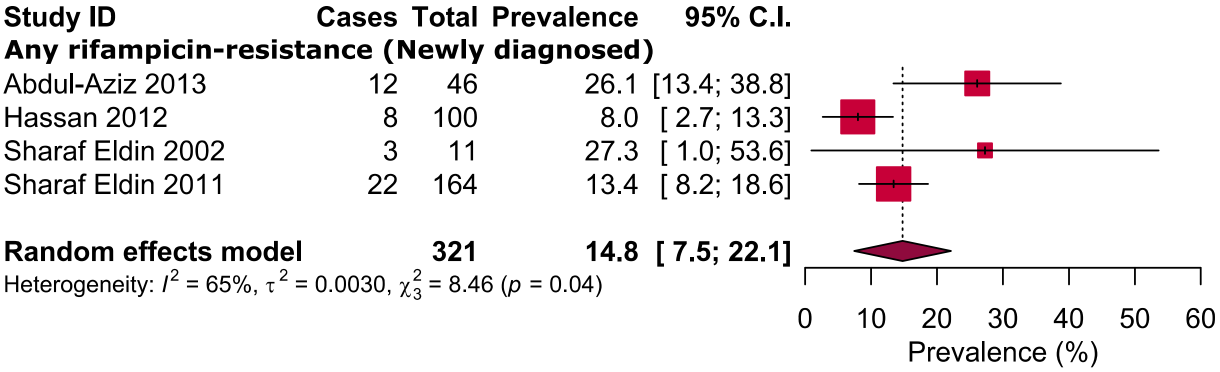

D

Study ID	Cases	Total	Prevalence	95% C.I.
**Any ethambutol-resistance (Newly diagnosed)**				
Abdul-Aziz 2013	4	46	8.7	[0.6; 16.8]
Hassan 2012	12	100	12.0	[5.6; 18.4]
Sharaf Eldin 2011	9	164	5.5	[2.0; 9.0]
**Random effects model**		**310**	**7.9**	**[3.8; 12.1]**

Heterogeneity: $I^2 = 38\%$, $\tau^2 = 0.0005$, $\chi^2_2 = 3.24$ ($p = 0.20$)

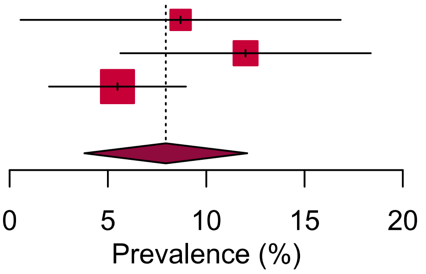

E

Study ID	Cases	Total	Prevalence	95% C.I.
**Any pyrazinamide-resistance (Newly diagnosed)**				
Hassan 2012	1	100	1.0	[0; 3]

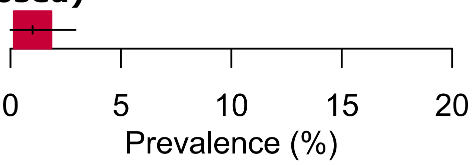

F

F

Study ID	Cases	Total	Prevalence	95% C.I.
**Any streptomycin-resistance (Previously treated)**				
Abdul-Aziz 2013	5	14	35.7	[10.6; 60.8]
Adam 2016	58	141	41.1	[33.0; 49.3]
Sharaf Eldin 2011	52	71	73.2	[62.9; 83.5]
**Random effects model**		**226**	**51.1**	**[26.1; 76.1]**

Heterogeneity: $I^2 = 92\%$, $\tau^2 = 0.0427$, $\chi^2_2 = 24.82$ ($p < 0.01$)

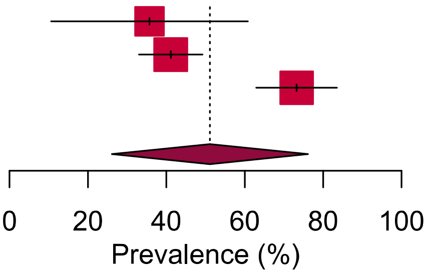

G

Study ID	Cases	Total	Prevalence	95% C.I.
**Any isoniazid-resistance (Previously treated)**				
Abdul-Aziz 2013	7	14	50.0	[23.8; 76.2]
Adam 2016	65	141	46.1	[37.9; 54.3]
Farah Aldour 2018	29	70	41.4	[29.9; 53.0]
Sharaf Eldin 2011	26	71	36.6	[25.4; 47.8]
**Random effects model**		**296**	**42.8**	**[37.2; 48.4]**

Heterogeneity: $I^2 = 0\%$, $\tau^2 = 0$, $\chi^2_3 = 2.13$ ($p = 0.55$)

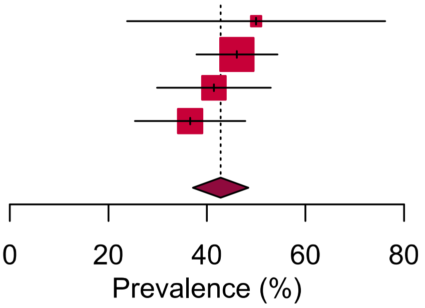

H

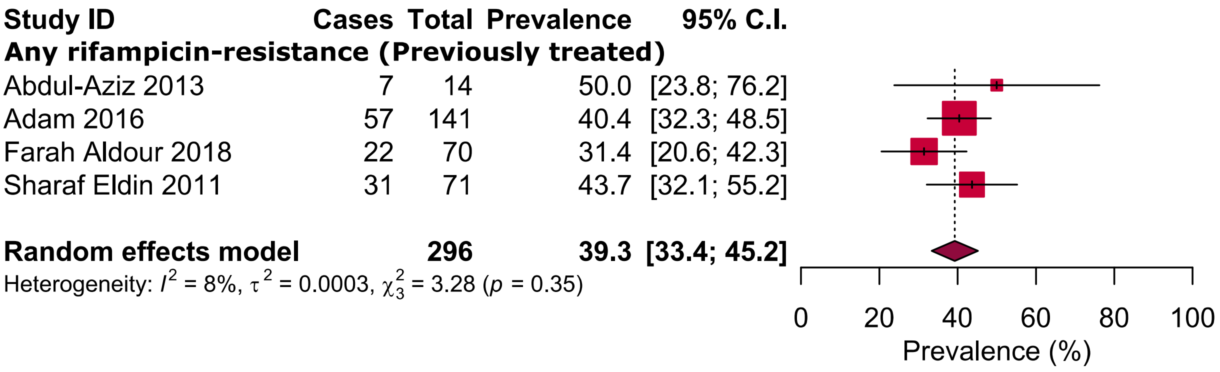

I

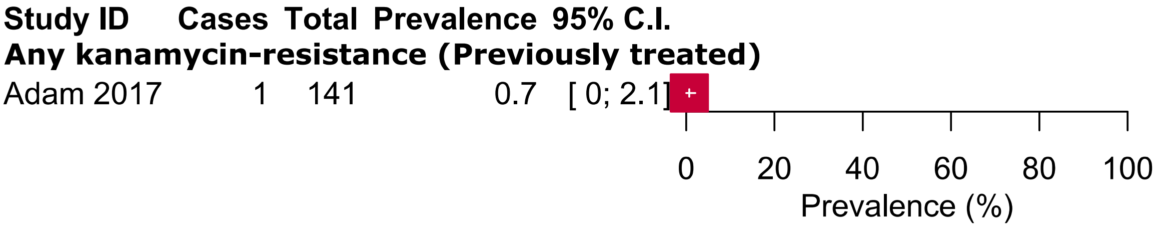

J

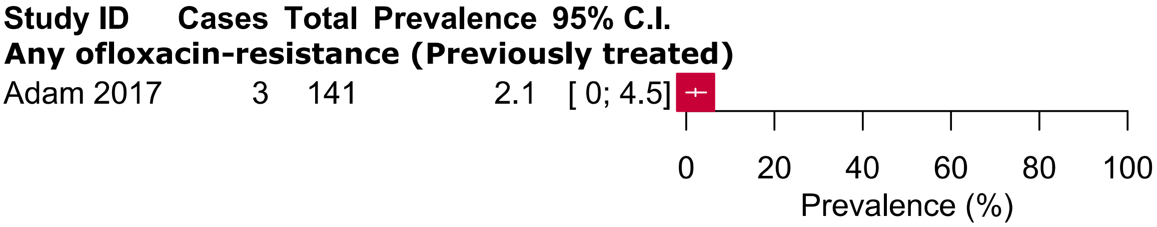

K

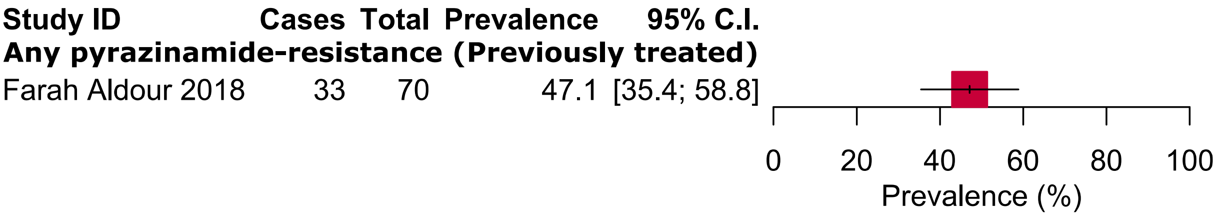

Figure S3. Any resistance to anti-TB drugs in newly diagnosed patients: A) streptomycin B) isoniazid, C) rifampicin, D) ethambutol, and E) pyrazinamide, and any resistance to anti-TB drugs in previously treated patients: F) streptomycin, G) isoniazid, H) rifampicin, I) kanamycin, J) ofloxacin, and K) pyrazinamide.

A

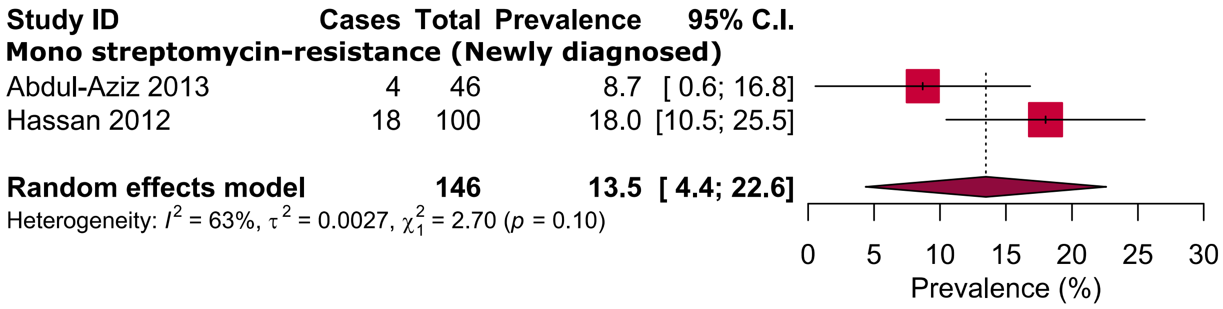
Study ID Cases Total Prevalence 95% C.I.
Mono streptomycin-resistance (Newly diagnosed)
Abdul-Aziz 2013 4 46 8.7 [0.6; 16.8]
Hassan 2012 18 100 18.0 [10.5; 25.5]
Random effects model 146 13.5 [4.4; 22.6]
Heterogeneity: I^2 = 63%, τ^2 = 0.0027, χ^2_1 = 2.70 (p = 0.10)
0 5 10 15 20 25 30
Prevalence (%)

B

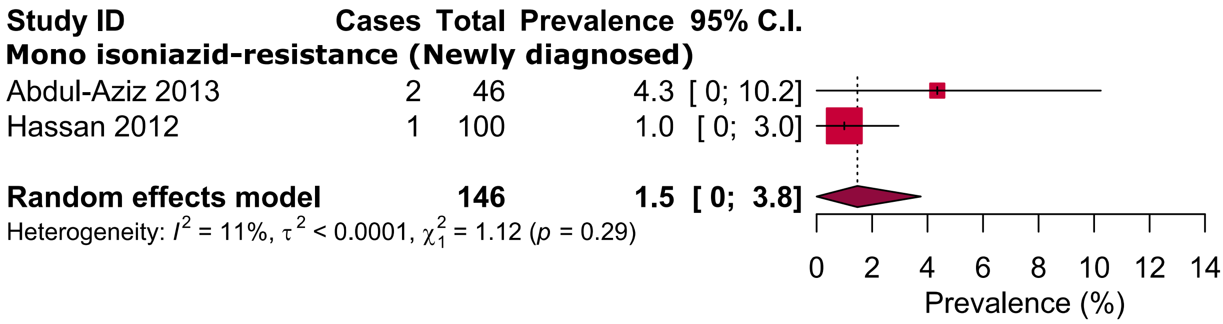
Study ID Cases Total Prevalence 95% C.I.
Mono isoniazid-resistance (Newly diagnosed)
Abdul-Aziz 2013 2 46 4.3 [0; 10.2]
Hassan 2012 1 100 1.0 [0; 3.0]
Random effects model 146 1.5 [0; 3.8]
Heterogeneity: I^2 = 11%, τ^2 < 0.0001, χ^2_1 = 1.12 (p = 0.29)
0 2 4 6 8 10 12 14
Prevalence (%)

C

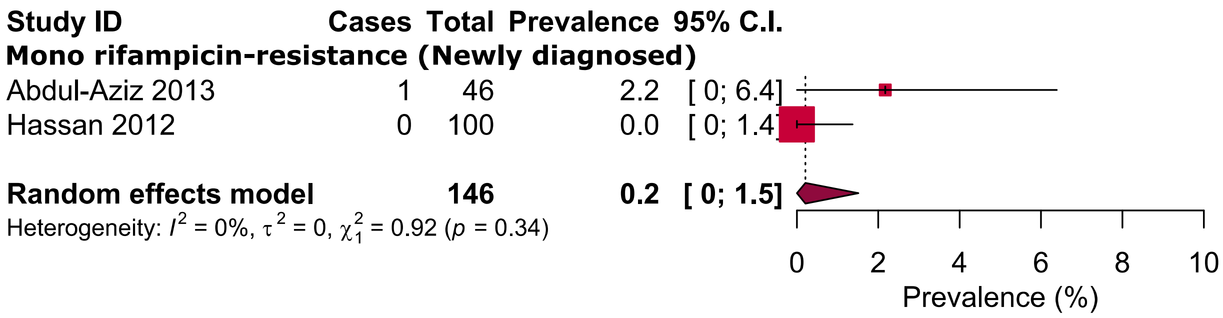
Study ID Cases Total Prevalence 95% C.I.
Mono rifampicin-resistance (Newly diagnosed)
Abdul-Aziz 2013 1 46 2.2 [0; 6.4]
Hassan 2012 0 100 0.0 [0; 1.4]
Random effects model 146 0.2 [0; 1.5]
Heterogeneity: I^2 = 0%, τ^2 = 0, χ^2_1 = 0.92 (p = 0.34)
0 2 4 6 8 10
Prevalence (%)

D

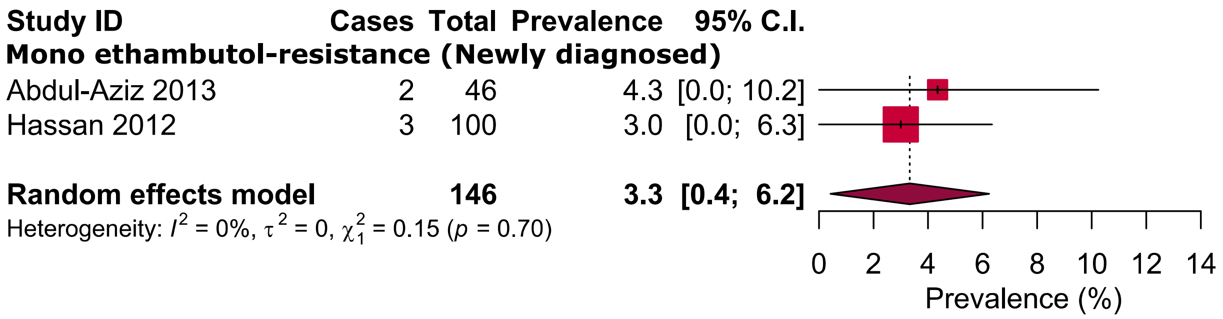
Study ID Cases Total Prevalence 95% C.I.
Mono ethambutol-resistance (Newly diagnosed)
Abdul-Aziz 2013 2 46 4.3 [0.0; 10.2]
Hassan 2012 3 100 3.0 [0.0; 6.3]
Random effects model 146 3.3 [0.4; 6.2]
Heterogeneity: I^2 = 0%, τ^2 = 0, χ^2_1 = 0.15 (p = 0.70)
0 2 4 6 8 10 12 14
Prevalence (%)

E

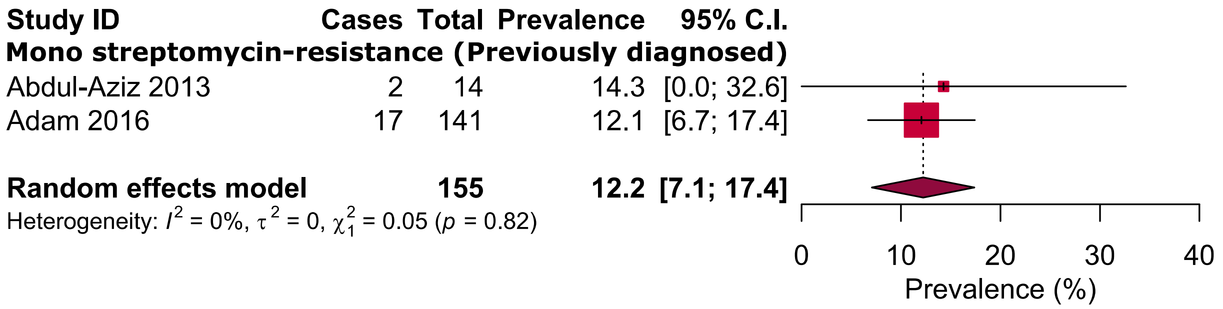

F

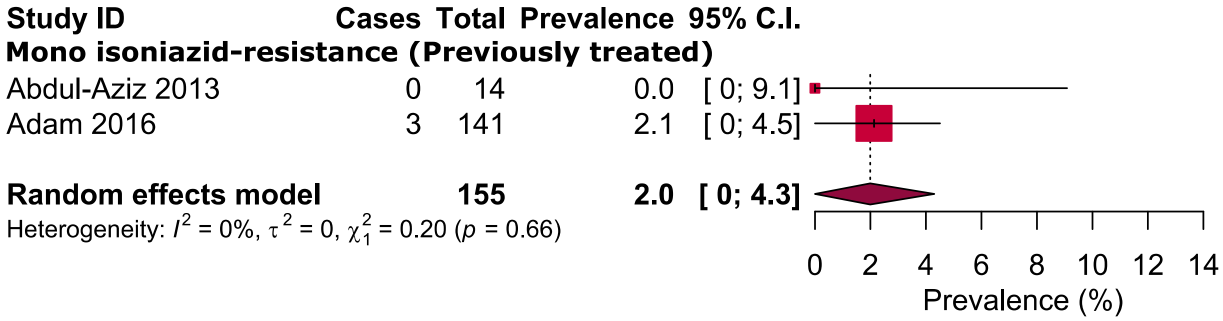

G

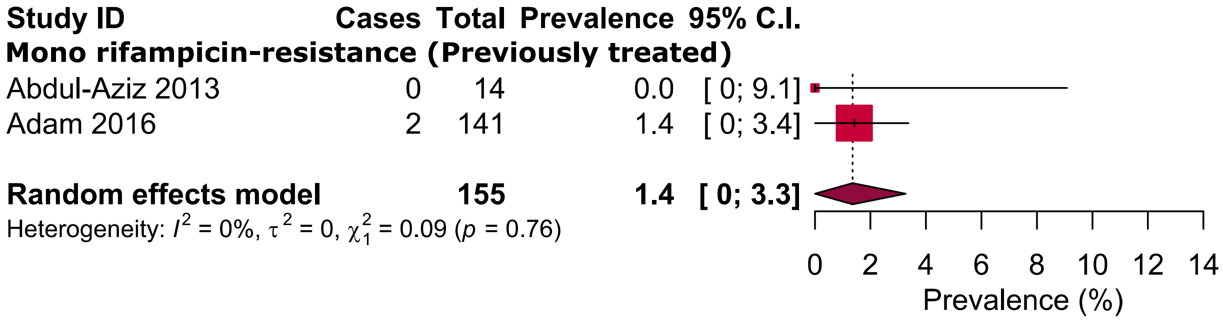

H

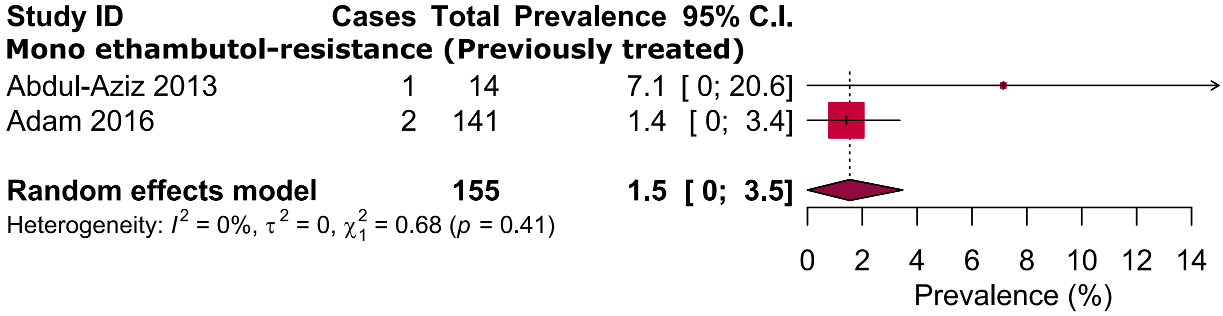

Figure S4. Mono resistance to anti-TB drugs in newly diagnosed patients: A) streptomycin, B) isoniazid, C) rifampicin, and D) ethambutol and mono resistance to anti-

TB drugs in previously treated patients: E) streptomycin, F) isoniazid, G) rifampicin, and H) ethambutol.

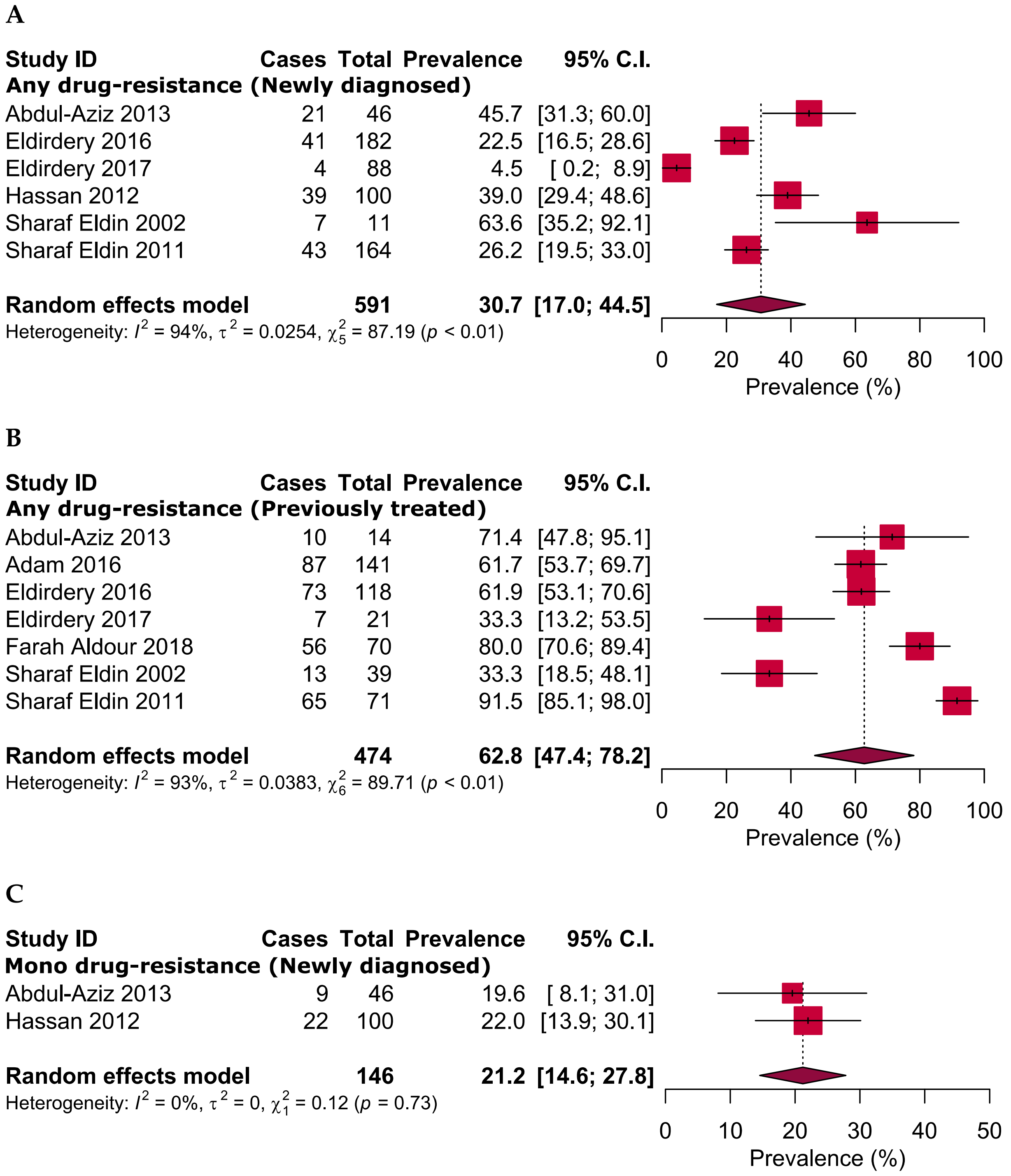

D

Study ID	Cases	Total	Prevalence	95% C.I.
**Mono drug-resistance (Previously treated)**				
Abdul-Aziz 2013	3	14	21.4	[0.0; 42.9]
Adam 2016	24	141	17.0	[10.8; 23.2]
Farah Aldour 2018	16	70	22.9	[13.0; 32.7]
**Random effects model**		**225**	**18.8**	**[13.7; 23.9]**

Heterogeneity: $I^2 = 0\%$, $\tau^2 = 0$, $\chi^2_2 = 1.03$ ($p = 0.60$)

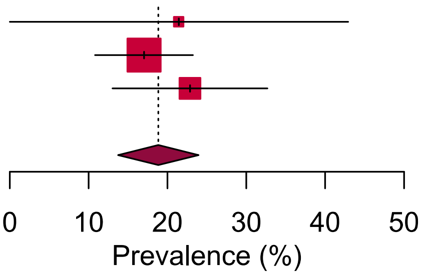

E

Study ID	Cases	Total	Prevalence	95% C.I.
**Multi drug-resistance (Newly diagnosed)**				
Abdul-Aziz 2013	10	46	21.7	[9.8; 33.7]
Ali 2017	4	25	16.0	[1.6; 30.4]
Eldirdery 2016	41	182	22.5	[16.5; 28.6]
Eldirdery 2017	4	88	4.5	[0.2; 8.9]
Hassan 2012	6	100	6.0	[1.3; 10.7]
Sharaf Eldin 2011	8	164	4.9	[1.6; 8.2]
**Random effects model**		**605**	**11.4**	**[5.2; 17.6]**

Heterogeneity: $I^2 = 86\%$, $\tau^2 = 0.0046$, $\chi^2_5 = 34.86$ ($p < 0.01$)

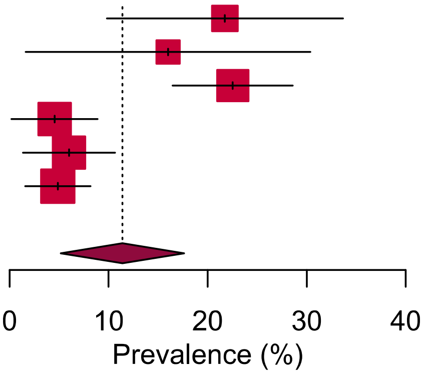

F

Study ID	Cases	Total	Prevalence	95% C.I.
**Multi drug-resistance (Previously treated)**				
Abdul-Aziz 2013	5	14	35.7	[10.6; 60.8]
Adam 2016b	54	141	38.3	[30.3; 46.3]
Ali 2017	37	72	51.4	[39.8; 62.9]
Eldirdery 2016	73	118	61.9	[53.1; 70.6]
Eldirdery 2017	7	21	33.3	[13.2; 53.5]
Farah Aldour 2018	29	70	41.4	[29.9; 53.0]
Sharaf Eldin 2011	17	71	23.9	[14.0; 33.9]
**Random effects model**		**507**	**41.5**	**[30.6; 52.5]**

Heterogeneity: $I^2 = 84\%$, $\tau^2 = 0.0170$, $\chi^2_6 = 36.58$ ($p < 0.01$)

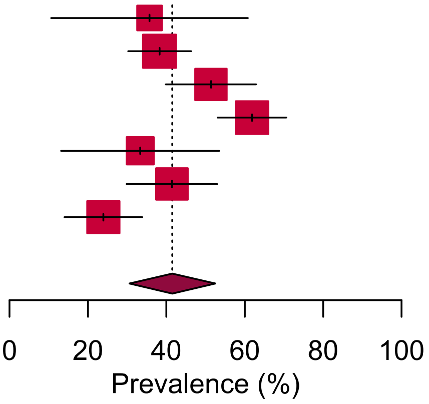

G

Study ID	Cases	Total	Prevalence	95% C.I.
**Poly drug-resistance (Newly diagnosed)**				
Abdul-Aziz 2013	1	46	2.2	[0; 6.4]

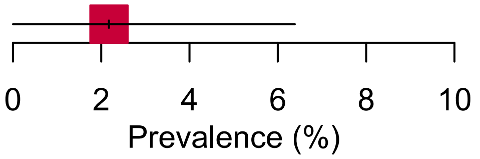

H

Study ID	Cases	Total	Prevalence	95% C.I.
**Poly drug-resistance (Previously treated)**				
Abdul-Aziz 2013	0	14	0.0	[0.0; 9.1]
Adam 2016	9	141	6.4	[2.3; 10.4]
Farah Aldour 2018	11	70	15.7	[7.2; 24.2]
**Random effects model**		**225**	**7.3**	**[0.0; 14.7]**

Heterogeneity: $I^2 = 69\%$, $\tau^2 = 0.0028$, $\chi^2_2 = 6.38$ ($p = 0.04$)

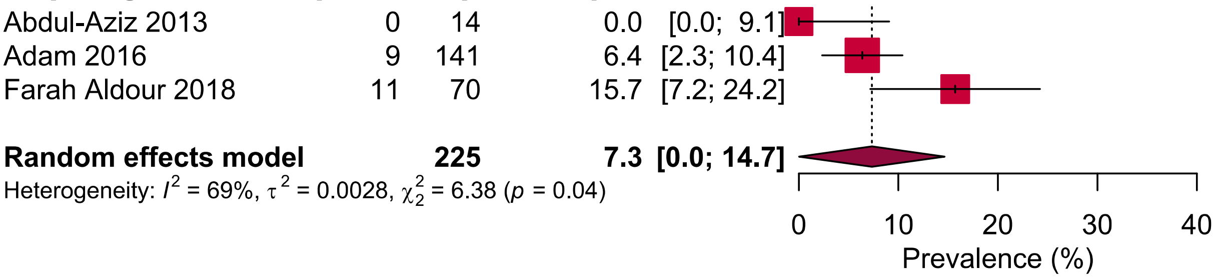

Figure S5. Overall drug resistance (DR) in newly diagnosed and previously treated TB patients. A) Any-DR in newly diagnosed patients, B) any-DR in previously treated patients, C) mono-DR in newly diagnosed patients, D) mono-DR in previously treated patients, E) multi-DR in newly diagnosed patients, F) multi-DR in previously treated patients, G) poly-DR in newly diagnosed patients, and H) poly-DR in previously treated patients.

A

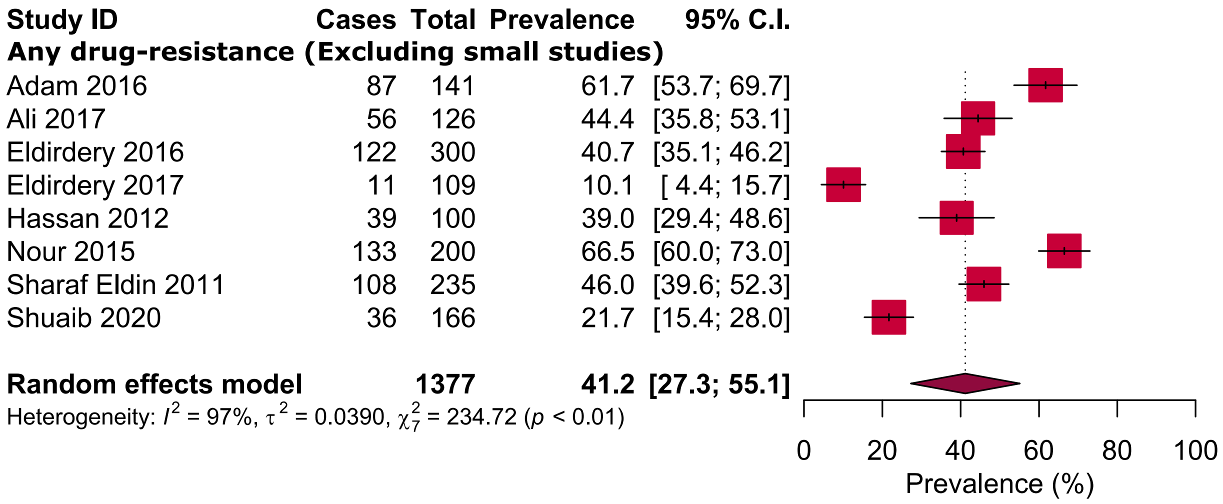
Study ID Cases Total Prevalence 95% C.I.
Any drug-resistance (Excluding small studies)
Adam 2016 87 141 61.7 [53.7; 69.7]
Ali 2017 56 126 44.4 [35.8; 53.1]
Eldirdery 2016 122 300 40.7 [35.1; 46.2]
Eldirdery 2017 11 109 10.1 [4.4; 15.7]
Hassan 2012 39 100 39.0 [29.4; 48.6]
Nour 2015 133 200 66.5 [60.0; 73.0]
Sharaf Eldin 2011 108 235 46.0 [39.6; 52.3]
Shuaib 2020 36 166 21.7 [15.4; 28.0]
Random effects model 1377 41.2 [27.3; 55.1]
Heterogeneity: I^2 = 97%, τ^2 = 0.0390, χ^2_7 = 234.72 (p < 0.01)
0 20 40 60 80 100
Prevalence (%)

B

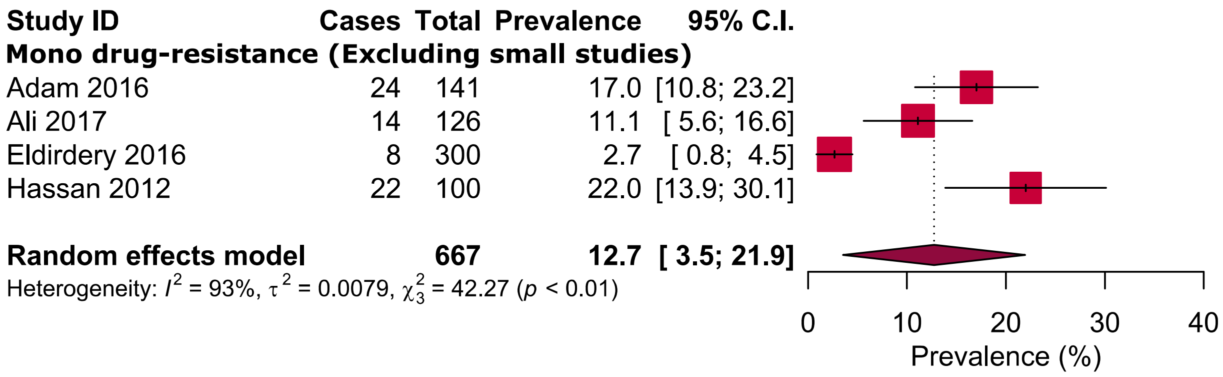
Study ID Cases Total Prevalence 95% C.I.
Mono drug-resistance (Excluding small studies)
Adam 2016 24 141 17.0 [10.8; 23.2]
Ali 2017 14 126 11.1 [5.6; 16.6]
Eldirdery 2016 8 300 2.7 [0.8; 4.5]
Hassan 2012 22 100 22.0 [13.9; 30.1]
Random effects model 667 12.7 [3.5; 21.9]
Heterogeneity: I^2 = 93%, τ^2 = 0.0079, χ^2_3 = 42.27 (p < 0.01)
0 10 20 30 40
Prevalence (%)

C

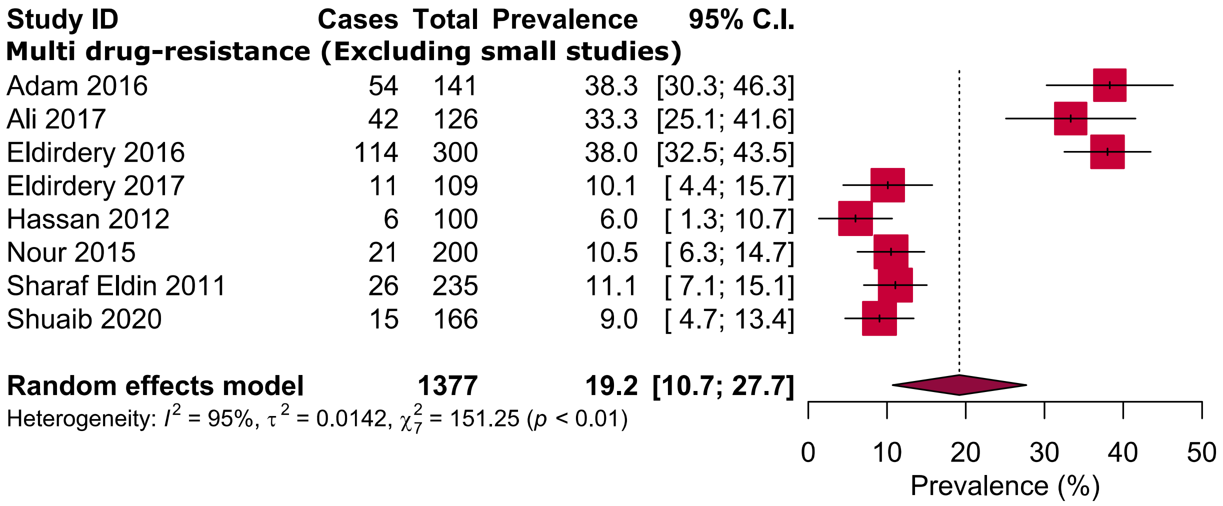
Study ID Cases Total Prevalence 95% C.I.
Multi drug-resistance (Excluding small studies)
Adam 2016 54 141 38.3 [30.3; 46.3]
Ali 2017 42 126 33.3 [25.1; 41.6]
Eldirdery 2016 114 300 38.0 [32.5; 43.5]
Eldirdery 2017 11 109 10.1 [4.4; 15.7]
Hassan 2012 6 100 6.0 [1.3; 10.7]
Nour 2015 21 200 10.5 [6.3; 14.7]
Sharaf Eldin 2011 26 235 11.1 [7.1; 15.1]
Shuaib 2020 15 166 9.0 [4.7; 13.4]
Random effects model 1377 19.2 [10.7; 27.7]
Heterogeneity: I^2 = 95%, τ^2 = 0.0142, χ^2_7 = 151.25 (p < 0.01)
0 10 20 30 40 50
Prevalence (%)

D

Study ID	Cases	Total	Prevalence	95% C.I.
**Poly drug-resistance (Excluding small studies)**				
Adam 2016	9	141	6.4	[2.3; 10.4]

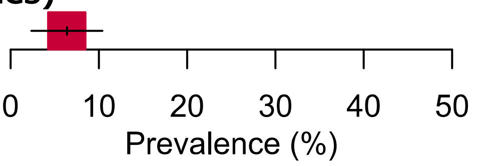

E

Study ID	Cases	Total	Prevalence	95% C.I.
**Extensively drug-resistance (Excluding small studies)**				
Adam 2017	1	141	0.7	[0; 2.1]

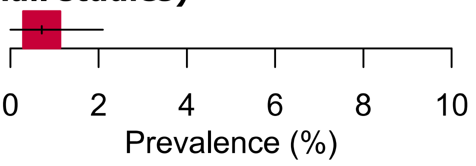

F

Study ID	Cases	Total	Prevalence	95% C.I.
**Any drug-resistance (Excluding moderate quality studies)**				
Abdul-Aziz 2013	31	60	51.7	[39.0; 64.3]
Adam 2016	87	141	61.7	[53.7; 69.7]
Ali 2017	56	126	44.4	[35.8; 53.1]
Eldirdery 2016	122	300	40.7	[35.1; 46.2]
Eldirdery 2017	11	109	10.1	[4.4; 15.7]
Elhassan 2012	38	56	67.9	[55.6; 80.1]
Hassan 2012	39	100	39.0	[29.4; 48.6]
Sharaf Eldin 2011	108	235	46.0	[39.6; 52.3]
**Random effects model**		**1127**	**44.9**	**[31.6; 58.1]**

Heterogeneity: $I^2 = 96\%$, $\tau^2 = 0.0344$, $\chi^2_7 = 162.81$ ($p < 0.01$)

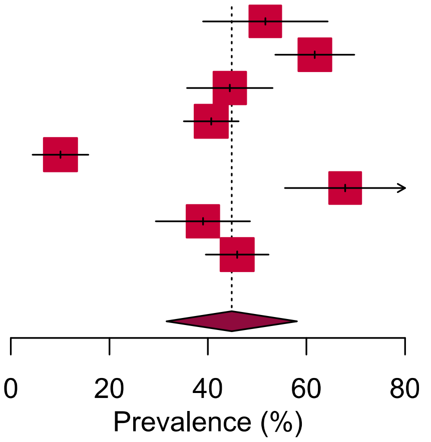

G

Study ID	Cases	Total	Prevalence	95% C.I.
**Mono drug-resistance (Excluding moderate quality studies)**				
Abdul-Aziz 2013	12	60	20.0	[9.9; 30.1]
Adam 2016	24	141	17.0	[10.8; 23.2]
Ali 2017	14	126	11.1	[5.6; 16.6]
Eldirdery 2016	8	300	2.7	[0.8; 4.5]
Elhassan 2012	8	56	14.3	[5.1; 23.5]
Hassan 2012	22	100	22.0	[13.9; 30.1]
**Random effects model**		**783**	**14.0**	**[6.4; 21.6]**

Heterogeneity: $I^2 = 91\%$, $\tau^2 = 0.0078$, $\chi^2_5 = 53.66$ ($p < 0.01$)

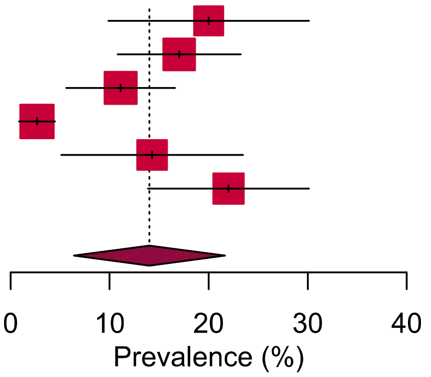

H

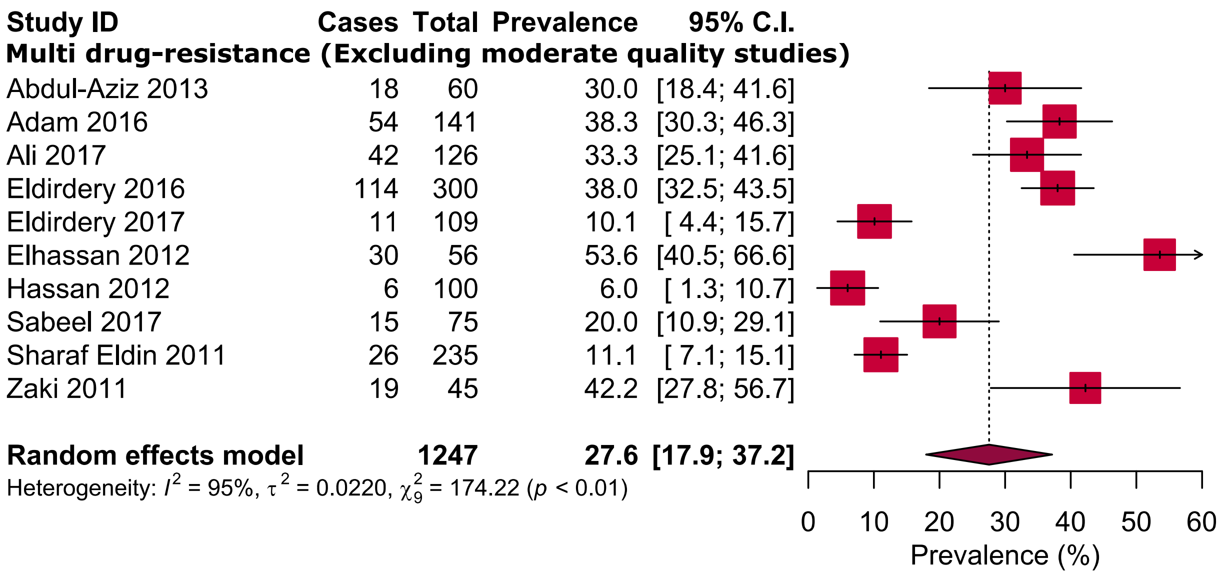
Study ID Cases Total Prevalence 95% C.I.
Multi drug-resistance (Excluding moderate quality studies)
Abdul-Aziz 2013 18 60 30.0 [18.4; 41.6]
Adam 2016 54 141 38.3 [30.3; 46.3]
Ali 2017 42 126 33.3 [25.1; 41.6]
Eldirdery 2016 114 300 38.0 [32.5; 43.5]
Eldirdery 2017 11 109 10.1 [4.4; 15.7]
Elhassan 2012 30 56 53.6 [40.5; 66.6]
Hassan 2012 6 100 6.0 [1.3; 10.7]
Sabeel 2017 15 75 20.0 [10.9; 29.1]
Sharaf Eldin 2011 26 235 11.1 [7.1; 15.1]
Zaki 2011 19 45 42.2 [27.8; 56.7]
Random effects model 1247 27.6 [17.9; 37.2]
Heterogeneity: I^2 = 95%, τ^2 = 0.0220, χ^2_9 = 174.22 (p < 0.01)
0 10 20 30 40 50 60
Prevalence (%)

I

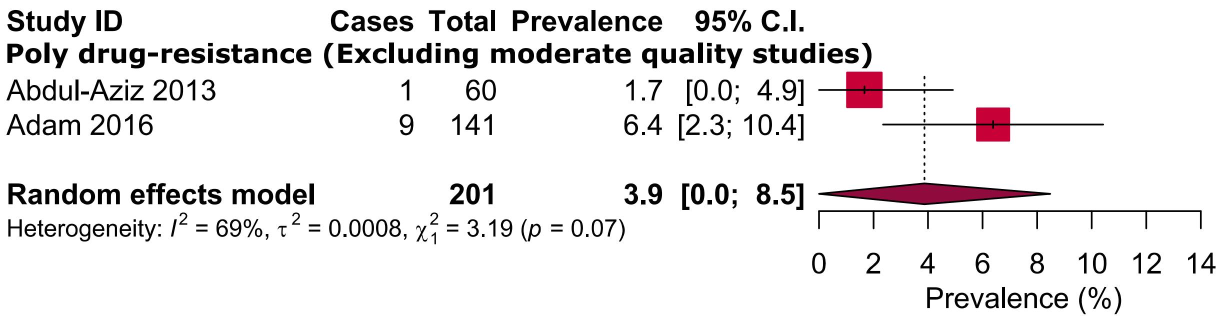
Study ID Cases Total Prevalence 95% C.I.
Poly drug-resistance (Excluding moderate quality studies)
Abdul-Aziz 2013 1 60 1.7 [0.0; 4.9]
Adam 2016 9 141 6.4 [2.3; 10.4]
Random effects model 201 3.9 [0.0; 8.5]
Heterogeneity: I^2 = 69%, τ^2 = 0.0008, χ^2_1 = 3.19 (p = 0.07)
0 2 4 6 8 10 12 14
Prevalence (%)

J

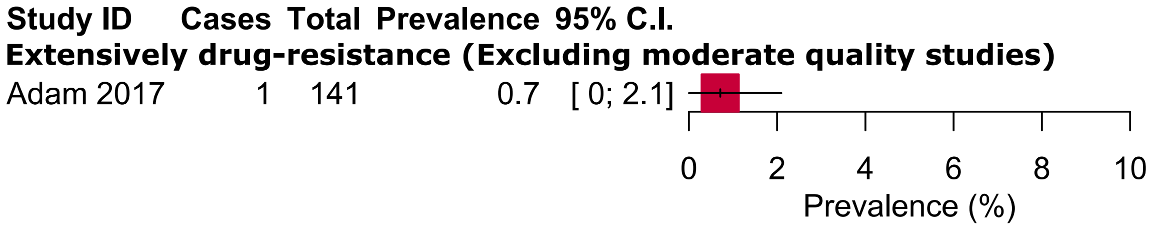
Study ID Cases Total Prevalence 95% C.I.
Extensively drug-resistance (Excluding moderate quality studies)
Adam 2017 1 141 0.7 [0; 2.1]
0 2 4 6 8 10
Prevalence (%)

K

Study ID	Cases	Total	Prevalence	95% C.I.
**Any drug-resistance (Using a fixed-effects model)**				
Abdul-Aziz 2013	31	60	51.7	[39.0; 64.3]
Adam 2016	87	141	61.7	[53.7; 69.7]
Ali 2017	56	126	44.4	[35.8; 53.1]
Eldirdery 2016	122	300	40.7	[35.1; 46.2]
Eldirdery 2017	11	109	10.1	[4.4; 15.7]
Elhassan 2012	38	56	67.9	[55.6; 80.1]
Farah Aldour 2018	56	70	80.0	[70.6; 89.4]
Hassan 2012	39	100	39.0	[29.4; 48.6]
Khalid 2015	23	53	43.4	[30.1; 56.7]
Nour 2015	133	200	66.5	[60.0; 73.0]
Sharaf Eldin 2002	20	50	40.0	[26.4; 53.6]
Sharaf Eldin 2011	108	235	46.0	[39.6; 52.3]
Shuaib 2020	36	166	21.7	[15.4; 28.0]
**Fixed effect model**		**1666**	**42.4**	**[40.2; 44.6]**
**Random effects model**			**47.0**	**[35.5; 58.6]**

Heterogeneity: $I^2 = 96\%$, $\tau^2 = 0.0428$, $\chi^2_{12} = 324.67$ ($p < 0.01$)

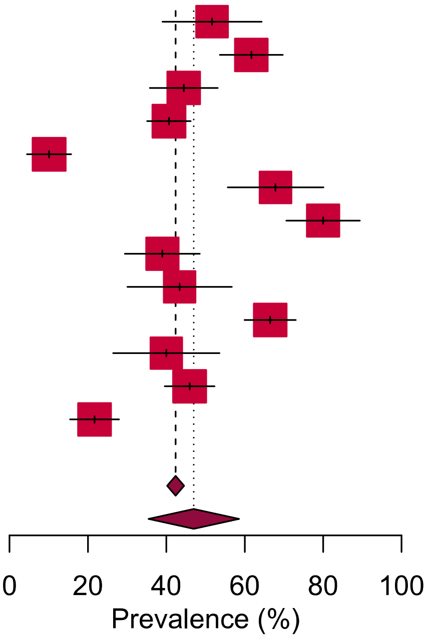

L

Study ID	Cases	Total	Prevalence	95% C.I.
**Mono drug-resistance (Using a fixed-effects model)**				
Abdul-Aziz 2013	12	60	20.0	[9.9; 30.1]
Adam 2016	24	141	17.0	[10.8; 23.2]
Ali 2017	14	126	11.1	[5.6; 16.6]
Eldirdery 2016	8	300	2.7	[0.8; 4.5]
Elhassan 2012	8	56	14.3	[5.1; 23.5]
Farah Aldour 2018	16	70	22.9	[13.0; 32.7]
Hassan 2012	22	100	22.0	[13.9; 30.1]
Sharaf Eldin 2002	12	50	24.0	[12.2; 35.8]
**Fixed effect model**		**903**	**6.6**	**[5.0; 8.1]**
**Random effects model**			**16.2**	**[9.0; 23.4]**

Heterogeneity: $I^2 = 90\%$, $\tau^2 = 0.0091$, $\chi^2_{7} = 73.36$ ($p < 0.01$)

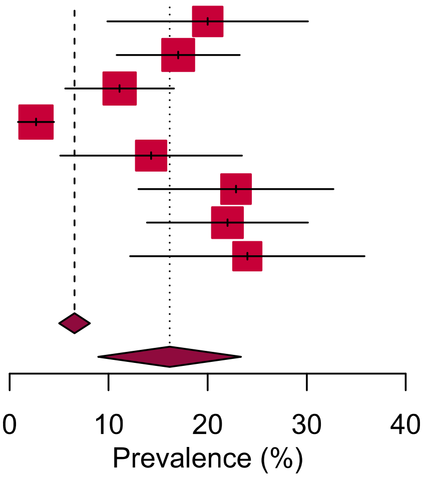

M

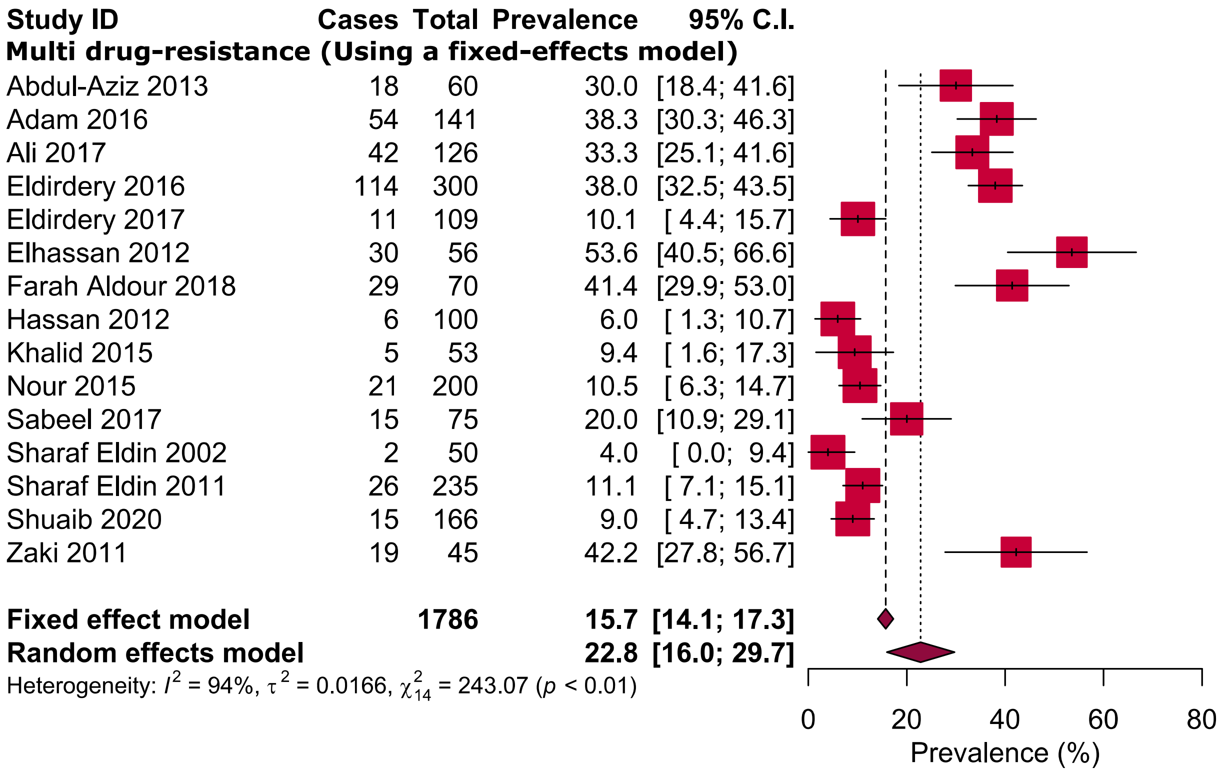

N

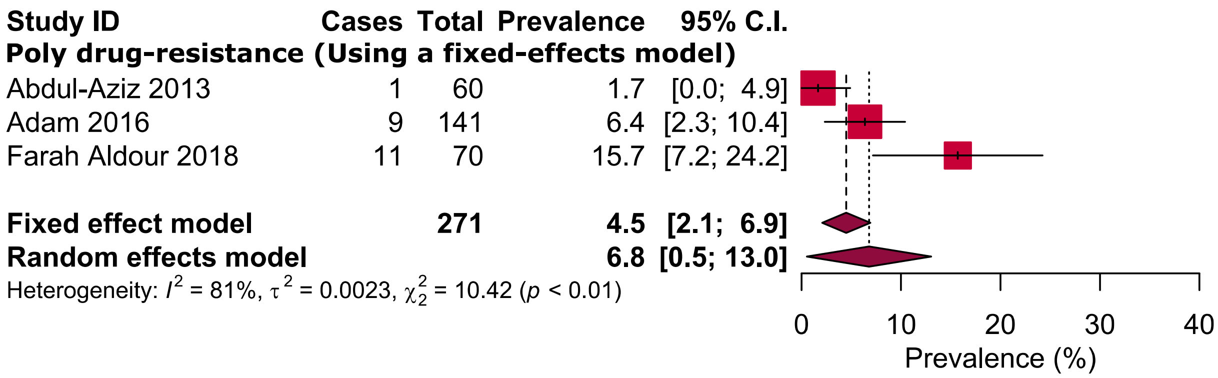

O

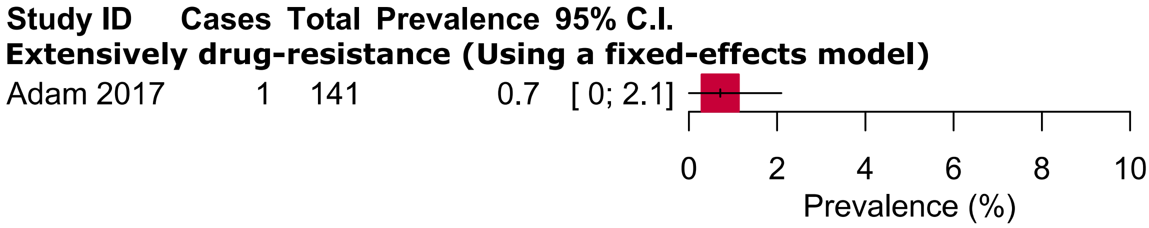

Figure S6. Sensitivity analyses: excluding small studies (n<100) from A to E; excluding moderate-quality studies from F to J and using a fixed-effects model from K to O.

Table S1. Quality assessment of the included cross-sectional studies.

No.	Study ID	Questions assessing included cross-sectional studies								Yes
		1	2	3	4	5	6	7	8	(%)
1	Abdul-Aziz 2013	Y	Y	Y	Y	N	N	Y	Y	75.0
2	Adam 2017	Y	Y	Y	Y	N	N	Y	Y	75.0
3	Adam 2016	Y	Y	Y	Y	N	N	Y	Y	75.0
4	Ali 2017	Y	Y	Y	Y	N	N	Y	Y	75.0
5	Eldirdery 2017	Y	Y	Y	Y	Y	N	Y	Y	87.5
6	Eldirdery 2016	Y	Y	Y	Y	N	N	Y	Y	75.0
7	Farah Aldour 2018	Y	Y	Y	U	N	N	Y	Y	62.5
8	Elhassan 2012	Y	Y	Y	Y	N	N	Y	Y	75.0
9	Hassan 2012	Y	Y	Y	Y	N	N	Y	Y	75.0
10	Khalid 2015	Y	Y	Y	Y	N	N	Y	N	62.5
11	Nour 2015	Y	Y	Y	Y	N	N	Y	N	62.5
12	Sabeel 2017	Y	Y	Y	Y	N	N	Y	Y	75.0
13	Sharaf Eldin 2011	Y	Y	Y	Y	Y	U	Y	Y	87.5
14	Sharaf Eldin 2002	Y	Y	Y	Y	N	N	Y	N	62.5
15	Shuaib 2020	Y	Y	Y	Y	N	N	N	N	50.0
16	Zaki 2011	Y	Y	Y	Y	Y	N	Y	N	75.0

1. Were the criteria for inclusion in the sample clearly defined? 2. Were the study subjects and the setting described in detail? 3. Was the exposure measured in a valid and reliable way? 4. Were objective, standard criteria used for measurement of the condition? 5. Were confounding factors identified? 6. Were strategies to deal with confounding factors stated? 7. Were the outcomes measured in a valid and reliable way? 8. Was appropriate statistical analysis used? Y=Yes; N=No; U=Unclear.

Table S2: Search strategy

Databases	Search strategy
**PubMed**	((TB OR tuberculosis OR "Mycobacterium tuberculosis" OR anti-tuberculosis OR anti-TB OR anti-tubercular) AND (isoniazid OR rifampicin OR ethambutol OR pyrazinamide OR streptomycin OR amikacin OR kanamycin OR capreomycin OR viomycin OR enviomycin OR ciprofloxacin OR levofloxacin OR moxifloxacin OR ethionamide OR prothionamide OR seromycin OR terizidone OR rifabutin

	OR clarithromycin OR linezolid OR thioacetazone OR bedaquiline OR clofazimine OR rifapentine OR resistance OR resistant OR susceptibility OR sensitivity OR drug-resistant OR drug resistance OR multidrug-resistant tuberculosis)) AND (Sudan OR Khartoum OR Kordofan OR Kassala OR Blue Nile OR Darfur OR Gezira OR White Nile OR River Nile OR Red Sea OR Al Qadarif OR Sennar)
**Scopus**	TITLE-ABS-KEY(TB OR tuberculosis OR "Mycobacterium tuberculosis" OR anti-tuberculosis OR anti-TB OR anti-tubercular) AND TITLE-ABS-KEY(isoniazid OR rifampicin OR ethambutol OR pyrazinamide OR streptomycin OR amikacin OR kanamycin OR capreomycin OR viomycin OR enviomycin OR ciprofloxacin OR levofloxacin OR moxifloxacin OR ethionamide OR prothionamide OR seromycin OR terizidone OR rifabutin OR clarithromycin OR linezolid OR thioacetazone OR Bedaquiline OR clofazimine OR rifapentine OR resistance OR resistant OR susceptibility OR sensitivity OR drug-resistant OR drug resistance OR multidrug-resistant tuberculosis) AND AFFIL(Sudan OR Khartoum OR Kordofan OR Kassala OR "Blue Nile" OR Darfur OR Gezira OR "White Nile" OR "River Nile" OR "Red Sea" OR "Al Qadarif" OR Sennar)
**Web of Science**	ALL=(TB OR tuberculosis OR “Mycobacterium tuberculosis” OR anti-tuberculosis OR anti-TB OR anti-tubercular) AND ALL=(isoniazid OR rifampicin OR ethambutol OR pyrazinamide OR streptomycin OR amikacin OR kanamycin OR capreomycin OR viomycin OR enviomycin OR ciprofloxacin OR levofloxacin OR moxifloxacin OR ethionamide OR prothionamide OR seromycin OR terizidone OR rifabutin OR clarithromycin OR linezolid OR thioacetazone OR bedaquiline OR clofazimine OR rifapentine OR resistance OR resistant OR susceptibility OR sensitivity OR drug-resistant OR drug resistance OR multidrug-resistant tuberculosis) AND ALL=(Sudan OR Khartoum OR Kordofan OR Kassala OR Blue Nile OR Darfur OR Gezira OR White Nile OR River Nile OR Red Sea OR Al Qadarif OR Sennar)
**Google Scholar**	allintitle:(TB OR tuberculosis OR “Mycobacterium tuberculosis” OR anti-tuberculosis OR anti-TB OR anti-tubercular) (Sudan OR Khartoum OR Kordofan

	OR Kassala OR "Blue Nile" OR Darfur OR Gezira OR "White Nile" OR "River Nile" OR "Red Sea" OR "Al Qadarif" OR Sennar)
